# *TP53I13* promotes metastasis in glioma *via* macrophages, neutrophils, and fibroblasts and is a potential prognostic biomarker

**DOI:** 10.3389/fimmu.2022.974346

**Published:** 2022-10-07

**Authors:** Xinqi Ge, Manyu Xu, Tong Cheng, Nan Hu, Pingping Sun, Bing Lu, Ziheng Wang, Jian Li

**Affiliations:** ^1^ Department of Clinical Biobank & Institute of Oncology, Affiliated Hospital of Nantong University, Medical School of Nantong University, Nantong, China; ^2^ Medical School of Nantong University, Nantong, China; ^3^ Department of Neurosurgery, Affiliated Hospital of Nantong University, Medical School of Nantong University, Nantong, China; ^4^ Centre for Precision Medicine Research and Training, Faculty of Health Sciences, University of Macau, Macau SAR, China

**Keywords:** TP53I13, TCGA, CGGA, CIBERSORT, IHC, immune infiltration, TME

## Abstract

**Background:**

TP53I13 is a protein coding tumor suppression gene encoded by the tumor protein p53. Overexpression of *TP53I13* impedes tumor cell proliferation. Nevertheless, *TP53I13* role and expression in the emergence and progression of glioma (low-grade glioma and glioblastoma) are yet to be identified. Thus, we aim to use comprehensive bioinformatics analyses to investigate TP53I13 and its prognostic value in gliomas.

**Methods:**

Multiple databases were consulted to evaluate and assess the expression of *TP53I13*, such as the Cancer Genome Atlas (TCGA), the Chinese Glioma Genome Atlas (CGGA), GeneMANIA, and Gene Expression Profiling Interactive. *TP53I13* expression was further explored using immunohistochemistry (IHC) and multiplex immunohistochemistry (mIHC). Through Gene Set Enrichment Analysis (GSEA), the biological functions of *TP53I13* and metastatic processes associated with it were studied.

**Results:**

The expression of *TP53I13* was higher in tumor samples compared to normal samples. In samples retrieved from the TCGA and CGGA databases, high *TP53I13* expression was associated with poor survival outcomes. The analysis of multivariate Cox showed that *TP53I13* might be an independent prognostic marker of glioma. It was also found that increased expression of *TP53I13* was significantly correlated with PRS type, status, 1p/19q codeletion status, IDH mutation status, chemotherapy, age, and tumor grade. According to CIBERSORT (Cell-type Identification by Estimating Relative Subsets of RNA Transcript), the expression of *TP53I13* correlates with macrophages, neutrophils, and dendritic cells. GSEA shows a close correlation between *TP53I13* and p53 signaling pathways, DNA replication, and the pentose phosphate pathway.

**Conclusion:**

Our results reveal a close correlation between *TP53I13* and gliomas. Further, *TP53I13* expression could affect the survival outcomes in glioma patients. In addition, *TP53I13* was an independent marker that was crucial in regulating the infiltration of immune cells into tumors. As a result of these findings, *TP53I13* might represent a new biomarker of immune infiltration and prognosis in patients with gliomas.

## Introduction

Gliomas are the most commonly occurring malignant adult brain tumors and include a diverse set of primary brain tumors like low-grade and high-grade gliomas (1–3). Glioblastomas account for 70~75% of all gliomas, while low-grade gliomas account for 20~25% of all gliomas (4, 5). Despite the low cases compared to glioblastomas, the low-grade gliomas can progress to glioblastomas and develop resistance to chemotherapy (6). Hence, low-grade gliomas could be lethal and malignant. Currently, multiple treatment strategies like chemotherapy, radiotherapy, and surgery are available that can improve the prognostic outcomes of glioma patients. However, the prognosis of gliomas is still grim, as the 1-year survival rate of glioma patients is inferior to 30%, and the 5-year progression-free survival (PFS) for World Health Organization (WHO) grade II and III gliomas (7, 8) is 50%. Various factors and mechanisms, including genetic and epigenetic alterations, contribute to the pathogenesis of glioma (9). Despite the efforts made to understand the mechanisms associated with glioma development, the molecular pathogenesis of gliomas remains unknown (10). Therefore, a comprehensive investigation of glioma pathogenesis and identifying critical biomarkers could be instrumental in accelerating and improving the diagnosis and treatment of gliomas.

A protein-coding gene called *TP53I13* is suspected to be a tumor suppressor. *TP53I1*3 overexpression is suggested to impede tumor cell growth. A report indicated that *TP53I13* expression could be upregulated by Adriamycin-induced genotoxic stress and/or p53/TP53-dependent ultraviolet irradiation (11). Interestingly, upregulated level of *TP53I13* helps to confirm that N4-Erucoyl spermidine could play a significant role in inhibiting hematological tumors (12). Therefore, an increase in *TP53I13* expression could impede tumor growth in hematological cancers. Based on previous studies, it is likely that overexpression of *TP53I13* in most normal tissues suppresses tumor formation. Therefore, it is compelling to postulate that a decrease in *TP53I13* levels could reduce the protection against tumors.

Gliomas, however, lack a clear understanding of the role of *TP53I13*. For this reason, data from CGGA (http://www.cgga.org.cn) and TCGA (https://tcga-data.nci) databases were used to investigate the role of *TP53I13* in gliomas. An analysis of bioinformatics revealed higher *TP53I13* expression in tumor tissues. Correlation analysis between *TP53I13* expression and patient survival revealed that *TP53I13* overexpression was related to poor patient survival. This result suggests that a low *TP53I13* level could indicate better survival outcomes. Therefore, we hypothesize that the occurrence and progression of glioma could be related to the high *TP53I13* expression. As a result, we used publicly available databases, like TCGA and CGGA, to investigate the correlations between several clinical parameters and *TP53I13* expression in this study. To further assess the fundamental mechanisms of *TP53I13* in glioma, we evaluated the relationships between lymphocytes and *TP53I13* expression in cells using TCGA, CGGA, and TIMER databases. In addition, multiplex immunohistochemistry (mIHC) was used to validate the results. A gene set enrichment analysis (GSEA), STRING, and GeneMANIA were used to investigate *TP53I13* functions in gliomas. Based on the results, *TP53I13* expression appears to be closely correlated with body metabolism and a number of important pathways. Accordingly, *TP53I13* plays a significant role in the development of gliomas, and it can be used as a biomarker for glioma prognosis prediction.

## Materials and methods

### Data acquisition

From the TCGA and CGGA databases, clinical and transcriptomic data of glioma patients were retrieved. Glioma RNAseq data (mRNA_seq325 and mRNA_seq693) was retrieved from CGGA database. From the CGGA database, 1018 glioma samples were retrieved (**Table 1**), and 696 samples were retrieved from TCGA database (**Table 2**) for further analysis.

**Table 1:** Correlation between TP53I13 expression and different clinical factors based on CGGA.

		Total (749)	low expression (374)	high expression (375)	χ^2^	P
PRS_type	Primary	502	263	239	3.948	0.139
	Recurrenrt	222	101	121		
	Secondary	25	10	15		
Grade	WHO II	218	160	58	107.726	< 0.001
	WHO III	240	133	107		
	WHO IV	291	81	210		
Gender	Male	442	229	213	1.519	0.218
	Female	307	145	162		
Radio_status	No	124	50	74	5.490	0.019
	Yes	625	324	301		
Chemo_status	No	229	131	98	6.977	0.008
	Yes	520	243	277		
IDH_mutation_status	Wildtype	339	95	244	118.913	< 0.001
	Mutant	410	279	131		
1p19q_codeletion_status	Non-codel	594	248	346	76.870	< 0.001
	Codel	155	126	29		

**Table 2:** The relationship between TP53I13 expression and different clinical factors based on TCGA.

Characteristic	levels	Low expression of TP53I13	High expression of TP53I13	p
n		348	348	
WHO grade, n (%)	G2	167 (26.3%)	57 (9%)	< 0.001
	G3	134 (21.1%)	109 (17.2%)	
	G4	17 (2.7%)	151 (23.8%)	
IDH status, n (%)	WT	32 (4.7%)	214 (31.2%)	< 0.001
	Mut	313 (45.6%)	127 (18.5%)	
1p/19q codeletion, n (%)	codel	149 (21.6%)	22 (3.2%)	< 0.001
	non-codel	198 (28.7%)	320 (46.4%)	
Primary therapy outcome, n (%)	PD	60 (13%)	52 (11.3%)	0.032
	SD	97 (21%)	50 (10.8%)	
	PR	36 (7.8%)	28 (6.1%)	
	CR	97 (21%)	42 (9.1%)	
Age, n (%)	<=60	312 (44.8%)	241 (34.6%)	< 0.001
	>60	36 (5.2%)	107 (15.4%)	
Histological type, n (%)	Astrocytoma	91 (13.1%)	104 (14.9%)	< 0.001
	Glioblastoma	17 (2.4%)	151 (21.7%)	
	Oligoastrocytoma	87 (12.5%)	47 (6.8%)	
	Oligodendroglioma	153 (22%)	46 (6.6%)	
OS event, n (%)	Alive	268 (38.5%)	156 (22.4%)	< 0.001
	Dead	80 (11.5%)	192 (27.6%)	
DSS event, n (%)	Alive	271 (40.1%)	160 (23.7%)	< 0.001
	Dead	71 (10.5%)	173 (25.6%)	
PFI event, n (%)	Alive	229 (32.9%)	121 (17.4%)	< 0.001
	Dead	119 (17.1%)	227 (32.6%)	
Age, median (IQR)		39 (31.75, 51)	53 (38.75, 63)	< 0.001

Tissues from 183 glioma patients were collected from Nantong University Affiliated Hospital (**Table 3**). Samples with missing information were removed. To study the relationships between the *TP53I13* expression and WHO grades, all tissues were divided into seven similar sets of tissue microarray chips (explained as tissue microarray chips 1~7, **Supplementary Table 1**). The independent prognostic value of *TP53I13* was investigated on samples obtained from CGGA and Nantong University Affiliated Hospital (**Supplementary Tables 2**-**3**).

**Table 3:** MIHC analysis between TP53I13 expression level and different clinical characteristics based on samples from Nantong University Affiliated Hospital.

Characteristic	Levels	TP53I13		Total	X^2^	P
		Low expression of TP53I13	High expression of TP53I13			
n		74	109			
Age	≤60	51 (40.8%)	74 (59.2%)	125	0.022	0.883
	>60	23 (39.7%)	35 (60.3%)	58
Gender	Female	33 (40.7%)	48 (59.3%)	81	0.006	0.941
	Male	41 (40.2%)	61 (59.8%)	102
IDH status	Wildtype	8 (26.7%)	22 (73.3%)	30	2.825	0.093
	Mutant	66 (43.1%)	87 (56.9%)	153
Type	AA, AG, and AGG	6 (54.5%)	5 (45.5%)	11	3.41	0.492
	AOA, A, DA, and OG	39 (40.2%)	58 (59.8%)	97
	GBM	14 (33.3%)	28 (66.7%)	42
	MG	10 (40.0%)	15 (60.0%)	25
	PA, PPXA, and PMA	5 (62.5%)	3 (37.5%)	8
WHO grade	G3	24 (51.1%)	23 (48.9%)	47	6.77	0.034
	G4	29 (31.2%)	64 (68.8%)	93
	G5	21 (48.8%)	22 (51.2%)	43		

### GEPIA

The Genotype-Tissue Expression (GTEx) high-throughput RNA sequencing data were analyzed and visualized using the Gene Expression Profiling Interactive Analysis (GEPIA) web-based bioinformatics tool (13, 14). *TP53I13* expression levels in tumor and normal samples were analyzed using GEPIA. Patient survival analysis based on *TP53I13* expression levels in glioma tissues was also provided by GEPIA.

### Immunohistochemistry

For tissue microarrays, the tissues were dewaxed, and antigen retrieval was performed. After eliminating endogenous enzymes in the tissues with 3% peroxidase solution, 5% bovine serum albumin was incubated for 20 minutes at room temperature. An overnight incubation with primary antibody was performed on the tissues. Following the primary antibody booster incubation, the tissues were incubated with the secondary antibody booster for 30 minutes. 3,3’-Diaminobenzidine was used to detect the tissues after 30 minutes of incubation with secondary antibody. After dehydrating, sealing, and observing the tissues, hematoxylin was applied to counterstain the tissues. The immunohistochemical staining was carried out on tissues obtained from the biological sample bank of Affiliated Hospital of Nantong University.

### Multiplex immunohistochemistry

mIHC employs chromogenic and fluorogenic methods, widely used in cancer immunology (15). mIHC was performed on tissue sections. The tissues were labeled with primary antibodies against TP53I13, CD68, CD163, CD66b, and S100A4, followed by incubation with suitable secondary antibodies. The details of all antibodies used are listed in **Supplementary Table 4**. The antigens were fixed by heating, followed by cooling, and tyramide signal amplification, which labeled the tissue section with fluorescent immunostains for each marker. For the evaluation and detection of the makers, an automated Vectra 3.0 quantitative pathology imaging system was utilized.

### LinkedOmics analysis

LinkedOmics (http://linkedomics.org/) is a novel and unique tool for inclusive analysis of all 32 TCGA cancer-related datasets (16). The web-based database can be used to generate plots for single genes, and the outcomes are displayed in the form of scatter plots, heatmaps, or volcano plots (17). In this study, the LinkedOmics platform was used to explore genes that correlated with *TP53I13 (both* negatively and positively) to determine the molecular mechanism associated with *TP53I13*.

### Protein-protein interaction analysis

An analysis of PPIs was carried out with the Search Tool for Retrieval of Interacting Genes (STRING) database (http://string-db.org). In order to identify hub genes, the PPI network model was visualized using Cytoscape software.

### Gene set enrichment analysis

An in-depth analysis of *TP53I13* biological functions was carried out using GSEA. C2.cp.kegg.v7.1.symbols.gmt was used for the Kyoto Encyclopedia of Genes and Genomes (KEGG) pathway enrichment analysis. C5.all.v7.4.symbols.gmt was selected for Gene Ontology enrichment analysis.

### Single-cell analysis of *TP53I13* expression levels in glioma

To further investigate the levels of *TP53I13* expression in glioma patients, we retrieved the GSE138749 dataset from the single cell TIME (scTIME) database (http://sctime.sklehabc.com/unicellular/home) and the GSE148842 dataset from the Tumor Immune Single-cell Hub (TISCH) (http://tisch.comp-genomics.org/) database. ScTIME includes 49 datasets, including information on 39 cancers for two species, humans and mice. scTIME also provides a series of single-cell analysis modules, including immune cell composition, correlation analysis of immune cell types, signature points specific to immune cell types, cell-cell communication, etc. In TISCH, cell types are categorized at the single-cell level, and TME is exploited in a wide range of cancers. 10X genomics was used to examine the data.

### Evaluation of Link between immune infiltration and *TP53I13* expression

The data obtained from TCGA and CGGA databases were analyzed using CIBERSORT, quanTiseq, xCell, and TIMER. To investigate the correlation between TP53I13 expression and immune infiltration, especially lymphocytic infiltration, CIBERSORT was employed to identify numerous immune infiltrating lymphocytes strongly correlated with *TP53I13* expression. To further validate the above analysis, we used quanTiseq, xCell, and TIMER. Infiltrating immune cells are identified by using the TIMER database (https://cistrome.shinyapps.io/timer/).

### Cell culture and transfection

Glioma cell lines U87 and U251 were used as *in vitro* models for analysis. Cell invasion, cell migration, and quantitative real-time polymerase chain reaction (qRT-PCR) were performed on these cells. Three different small interfering RNA (siRNA) sequences targeting *TP53I13* (siRNA 1-3) and NC (siRNA-NC) were designed using Invitrogen’s online software BLOCK-iTTM RNAi Designer and synthesized by Oligobio (OLIGOBIO, Beijing, China). Cells were transfected with Lipofectamine™ 3000 transfection reagent (Invitrogen, Carlsbad, USA). The siRNA sequence with the highest efficiency to interfere with the *TP53I13* expression was selected for further analysis.

The siRNA target sequences are as follows:

TP53I13 siRNAs: TP53I13 si1: GGGAATCCCTGGTAGGGAGAGTAAT, TP53I13 si2 GGAATCCCTGGTAGGGAGAGTAATG, and TP53I13 si3 GGCTGTGTCTGTTCAAGTCAGGCTT.

### Transwell assay

A transwell assay was used to test U-87’s and U-251’s migration and invasion abilities. Briefly, 5 × 10 (4) cells were seeded on chambers coated (for invasion) or uncoated with Matrigel (BD Biosciences, San Jose, CA) (for migration). Lower chambers were added DMEM medium containing serum and upper chambers were filled with serum-free medium. A 24 hour incubation was followed by the fixation of the cells with 4% paraformaldehyde and staining with 0.1% crystal violet. Cell counts were observed under light microscope.

## Results

### Relationship between *TP53I13* expression and TME in glioma patients

**Figure 1:**
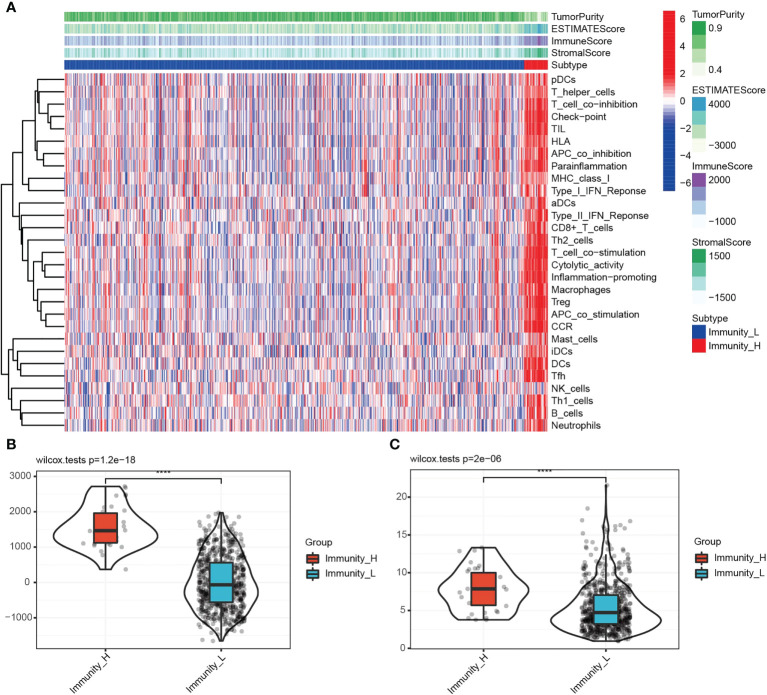
**(A)** Immune cell score, stromal cell score, combined scores of immune and stromal cells, and tumor purity in the high- and low-immunity groups. **(B)** The relationships between the immune score and different immunity groups. **(C)** The differences in *TP53I13* expression in the high- and low-immunity groups.

TME includes various cells and extracellular components, which significantly affect the immunotherapeutic response and clinical outcomes (18, 19). Stremitzer et al. (2020) have identified a significant relationship between tumor immune microenvironment and patient survival (20). In this study, Expression Data (ESTIMATE: R package) and CIBERSORT were used to score the tumor purity, interstitial cells and immune cells of TCGA glioma samples. According to the “PAM” method, samples were divided into groups with low and high immunity levels. Low immune group members had significantly lower ESTIMATE, immune and stoma scores than high immune group members. As compared to the hypoimmune group, tumor purity was lower in the high immune group (P=0.001). **Figures 1A, B** shows this. A significant connection was observed between immune cells and the high-expression group, indicating a correlation between the high-expression group, tumor microenvironment, and tumor-infiltrating immune cells. The relationship between *TP53I13* expression levels in high- and low-immunity groups was assessed, and high *TP53I13* expression was observed in the high-immunity group compared to the low-immunity group, consistent with TME analysis of glioma data (**Figure 1C**). *TP53I13* expression is related to the immune microenvironment of gliomas, as shown by the findings.

### *TP53I13* expression in various tumor types

**Figure 2:**
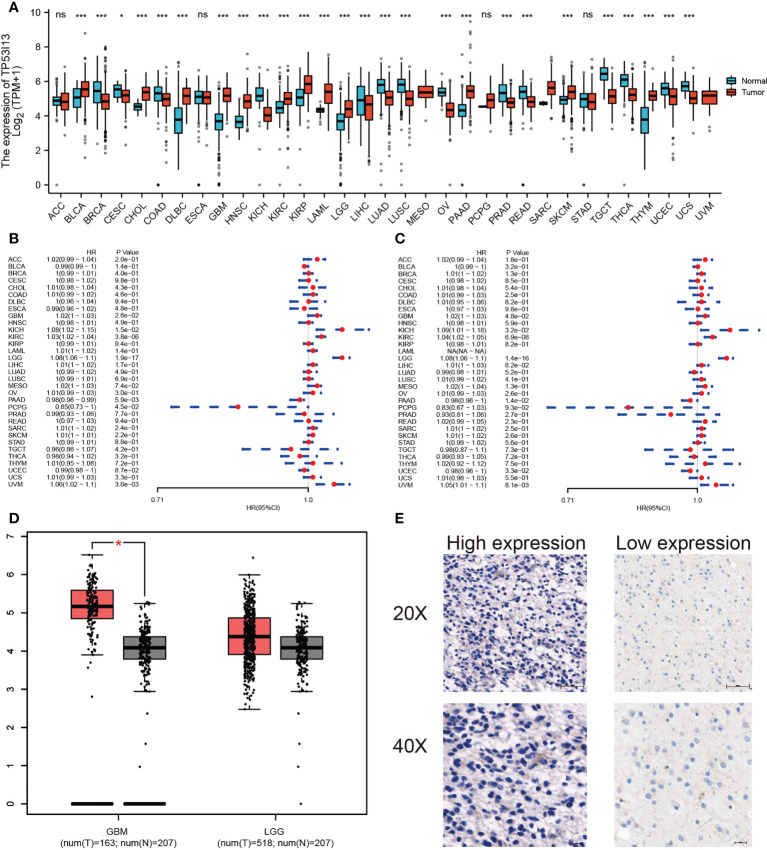
**(A)** Pan-cancer analysis of *TP53I13* in different tumors based on GTEx and TCGA databases *, P<0.05; ***, P<0.001. **(B)** The hazard ratio for overall survival in 33 tumors expressing *TP53I13*. **(C)** The hazard ratio for disease-specific survival in 33 tumors expressing *TP53I13*. **(D)** *TP53I13* expression based on the GEPIA database. **(E)** Representative immunohistochemistry (IHC) analysis shows high and low TP53I13 expression. ns, no significance.

*TP53I13* expression in various tumors and neighboring tissues were retrieved from GTEx and TCGA databases. On the other hand, tumor tissues expressed *TP53I13* at a higher level than normal tissues. The expression of *TP53I13* was detected in various cancer types such as glioblastoma tissues (**Figure 2A**), cholangiocarcinoma (CHOL), lymphoid neoplasm diffuse large B-cell lymphoma (DLBC), Kidney renal clear cell carcinoma (KIRC), skin cutaneous melanoma (SKCM), low-grade glioma (LGG), bladder urothelial carcinoma (BLCA), and thymoma (THYM). Survival analysis revealed overall survival (OS) was good in *TP53I13* expressing tumors including Pancreatic ductal adenocarcinoma (PAAD; P = 5.9e-03) and Pheochromocytoma and paraganglioma (PCPG; P = 4.5e-02). On the contrary, poor survival was observed *TP53I13* expressing tumors including like Uveal Melanoma (UVM; P = 3.6e-03), LGG (P = 1.9e-17), KIRC (P = 3.8e-06), kidney chromophobe (KICH; P = 1.5e-02), and glioblastoma (GBM; P = 2.6e-02) (**Figure 2B**).

*TP53I13* expression in cancers like PAAD (P = 1.4e-02) and uterine corpus endometrial carcinoma (UCEC; P = 3.3e-02) had good disease-specific survival (DSS). *TP53I13* expression in cancers like GBM (P = 4.8e-02), KICH (P = 3.2E-02), KIRC (P = 6.9e-08), LGG (P = 1.4e-16), and UVM (P = 8.1e-03) had poor DSS (**Figure 2C**). The intersection survival analysis of DSS and OS revealed that *TP53I13* has prognostic value in GBM, KICH, KIRC, LGG, and UVM.

### Prognostic value of *TP53I13* expression in glioma

**Figure 3:**
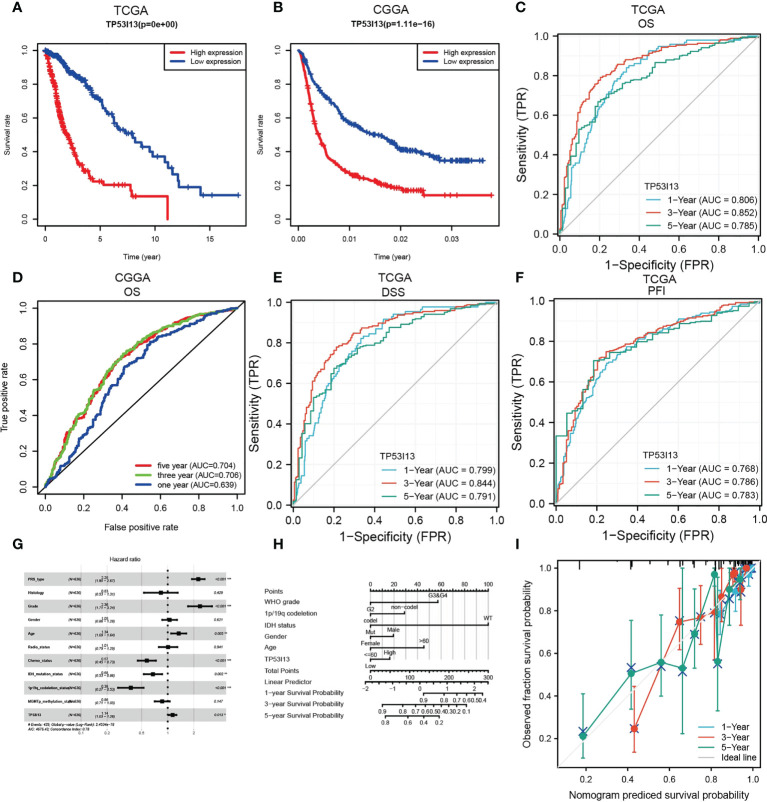
**(A)** Kaplan–Meier curve for overall survival (OS) of glioma patients with the TP53I13expression based on the TCGA database. **(B)** Kaplan–Meier curve of the OS of glioma patients with TP53I13 expression level based on the CGGA database. ROC curves for 1-, 3-, and 5-year OS analysis based on *TP53I13* expression levels in glioma patient samples obtained from **(C)** TCGA **(D)** CGGA databases. **(E)** ROC curves for 1-, 3-, and 5-year DSS analysis of TP53I13 expression in glioma patient samples obtained from TCGA. **(F)** ROC curves for 1-, 3-, and 5-year PFI analysis of *TP53I13* expression glioma patient samples obtained from TCGA. **(G)** Multivariate Cox regression analysis of the TP53I13 expression and clinical characteristics. **(H)** Nomogram for OS integrating *TP53I13* expression level, WHO grade, 1p/19q codeletion status, IDH mutation status, sex, and age. **(I)** The calibration curve for predicting 1-, 3-, and 5-year OS in glioma patients.

*TP53I13* expression was assessed in various tumors. Gliomas like LGG and GBM exhibit significant expression of TP53I13. To learn more about *TP53I13*’s prognostic value in gliomas, we conducted our study. GEPIA was used to investigate *TP53I13* expression, and the results reveal that *TP53I13* expression was low in normal tissues compared to gliomas such as LGG and GBM (**Figure 2D**). Similar results were obtained using immunohistochemical analysis (**Figure 2E**). Further, *TP53I13* expression was studied in tissue samples obtained from Nantong University Affiliated Hospital, and the results revealed that TP53I13 expression was higher in tumor tissues than in normal tissues (**Supplementary Figure 1A**). A TCGA and CGGA database containing glioma samples was examined for *TP53I13* mRNA expression. As shown in **Figures 3A, B**, according to the TCGA and CGGA data, high levels of *TP53I13* were associated with lower overall survival (OS). Similar results were obtained in glioma patient samples from Nantong University Affiliated Hospital, where high *TP53I13* expression was associated with poor survival (**Supplementary Figure 1B**). TP53I13 was evaluated for its ability to predict one-, three-, and five-year survival using ROC curves. TCGA’s data on 1-, 3-, and 5-year survival rates for glioma patients showed AUCs of 0.806, 0.852, and 0.785, respectively (**Figure 3C**). One-, three-, and five-year survival rates for CGGA in glioma patients were 0.704, 0.706, and 0.639, respectively (**Figure 3D**). We evaluated TP53I13’s prognostic value in glioma patient samples retrieved from TCGA regarding 1-year survival, 3-year survival, and 5-year survival. AUCs for one-year, three-year, and five-year DSS rates for glioma patients were 0.799, 0.844, and 0.791, respectively (**Figure 3E**). One-, three-, and five-year PFI survival rates of glioma patients were 0.768, 0.786, and 0.783 respectively, according to the AUC (**Figure 3F**). Furthermore, prognostic variables were assessed with Cox regression analysis. Multivariate Cox analysis identified low PRS type, low grade, less age, high 1p/19q codeletion status, high chemotherapy, high IDH mutation level, and low *TP53I13* expression as independent prognostic factors that predicted OS (**Figure 3G**). We also investigated the independent prognostic value of *TP53I13* on 159 glioma samples obtained from the Affiliated Hospital of Nantong University. A combination of WHO grade, age, sex, and *TP53I13* was an independent prognostic factor in glioma patients, based on the results of the study (**Supplementary Figure 1C**). In order to improve survival prediction mapping for glioma patients, we integrated *TP53I13* expression levels with other prognostic factors (**Figure 3H**). The nomograms were also calibrated to determine their accuracy. **Figure 3I** show that the curves showed good consistency with the predicted results.

### *TP53I13* expression in different subgroups of patients with glioma

**Figure 4:**
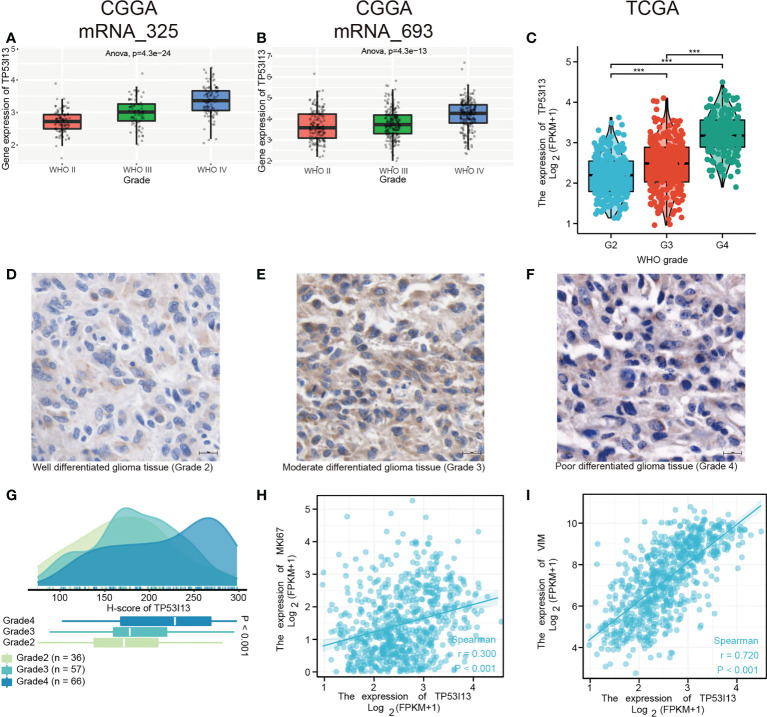
TP53I13 Expression in subgroups of glioma patients based on IDH mutation status, WHO grade, and 1p/19q codeletion status. Boxplot shows a correlation between *TP53I13* expression and WHO grade based on **(A)** the CGGA mRNAseq_325 dataset, **(B)** the CGGA mRNAseq_693 dataset, and **(C)** the TCGA database. **(D-F)** IHC of *TP53I13* in glioma tissues of different WHO grades. **(G)** Quantification of *TP53I13* staining in glioma tissues of different WHO grades. **(H)** The relationship between *TP53I13* and a proliferation marker (Ki-67). **(I)** The relationship between *TP53I13* and invasion markers (vimentin).

We analyzed the CGGA and TCGA databases for relationships between *TP53I13* expression and glioma subgroups. Additionally, the expression of TP53I13 was examined in patients with distinct WHO grades and codeletions of 1p/19q, as well as patients with IDH mutations. From the CGGA database, two datasets were analyzed, mRNAseq_325 and mRNAseq_693. According to CGGA and TCGA samples, TP53I13 expression increased as WHO grade increased (**Figures 4A-C**). Similar results were observed using IHC, where an increase in *TP53I13* expression was observed with an increase in WHO grade (**Figures 4D-F**). Further, seven tissue microarrays were carried out using the same method under the same experimental conditions, which included tissue samples from all the glioma patients for further analysis. Higher tumor grades had a worse prognosis, and *TP53I13* expression increased as the glioma progressed in all the glioma patients (**Figure 4G**). We further investigated the relationships between TP53I13, MKI67, and vimentin (VIM) expression levels. **Figure 4H** shows poor correlation between*TP53I13* and MKI67 (Ki-67 proliferation index) (R = 0.300, P <0.001) but *TP53I13* had a significant correlation with VIM (vimentin invasion index) (R = 0.720, P<0.001). (**Figure 4I**). The results reveal that enhanced *TP53I13* expression was linked to malignant clinicopathological features in glioma patients.

According to CGGA and TCGA glioma patient samples, IDH wild-type patients expressed higher levels of TP53I13 than IDH mutant patients (**Supplementary Figures 2A-C**). TP53I13 expression was also reduced in patients with 1p/19q codeletions compared with patients without (**Supplementary Figures 2D-F**).

### Multifactorial survival analysis of *TP53I13* expression

**Figure 5:**
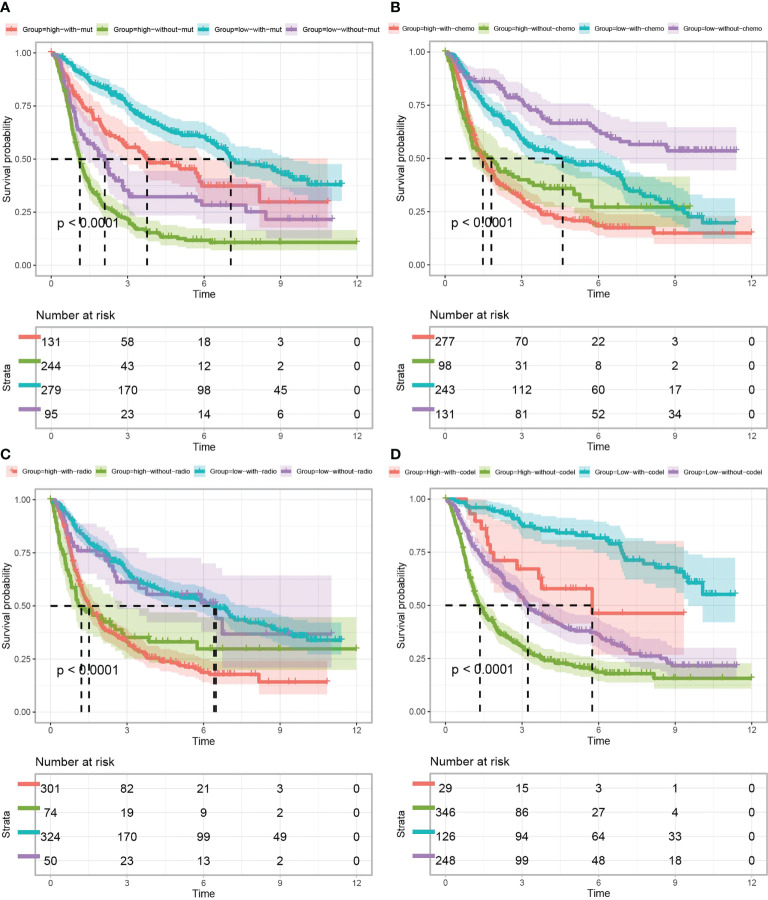
Kaplan–Meier curves of glioma patients (data obtained from CGGA) classified based on *TP53I13* expression and **(A)** IDH mutation, **(B)** chemotherapy, **(C)** radiotherapy, and **(D)** 1p/19q codeletion status.

Survival analysis between *TP53I13* expression and *IDH* mutation (**Figure 5A**), chemotherapy (**Figure 5B**), 1p/19q codeletion status (**Figure 5D**), and radiotherapy (**Figure 5C**) was performed. As shown in **Figure 5A**, patients harboring *IDH* mutation expressed high *TP53I13* levels (red and green) and had poor survival outcomes, further confirming that TP53I13 could be a potential prognostic biomarker for gliomas. As shown in **Figure 5B**, in glioma patients with high TP53I13 expression (red and green), poorer survival outcomes were observed similarly to IDH mutation status. As shown in **Figure 5C**, high *TP53I13* expression (red and green) was related to a poor survival outcome. Taking these results together, it appears that glioma patients with low TP53I13 expression could have a higher survival rate. As shown in **Figure 5D**, the patients with 1p/19q codeletion expressed a high level of TP53I13 and had poor survival outcomes.

### Prognostic value of *TP53I13* in glioma patients

Further study of the potential prognostic value of TP53I13 in gliomas, a survival analysis including OS and DSS was conducted on patients divided based on their clinical characteristics. Overall survival (OS) analysis revealed significant correlation between high *TP53I13* expression in females (P < 0.001) (**Supplementary Figure 3G**), males (P < 0.001) (**Supplementary Figure 3H**), patients without 1p/19 co-deletion (P < 0.001) (**Supplementary Figure 3F**), patients harboring IDH mutation (P = 0.028) (**Supplementary Figure 3D**), WHO grades 3 and 4 (P < 0.001), and WHO grade 2 (P = 0.038) (**Supplementary Figure 3A**), (**Supplementary Figure 3B**), and poor survival outcome. The results of the DSS analysis were consistent with the OS outcomes. Significant correlation was observed between high *TP53I13* expression and WHO grade 2 (P = 0.031) (**Supplementary Figure 4A**), WHO grades 3 and 4 (P < 0.001) (**Supplementary Figure 4B**), patients harboring IDH mutation (P = 0.024) (Supplementary **Figure 4D**), patients without 1p/19 co-deletion (P < 0.001) (**Supplementary Figure 4F**), females (**Supplementary Figure 4G**), and male (**Supplementary Figure 4H**) and poor survival outcomes. Patients with *TP53I13* show better prognosis than those with IDH mutations, WHO grades, or 1p/19q codeletion status. Therefore, these results suggest that TP53I13 can be used as a biomarker for predicting glioma and can predict survival outcomes in patients with glioma.

### Investigating differentially expressed genes (DEmRNAs, DElncRNAs, and DEmiRNAs)

**Figure 6:**
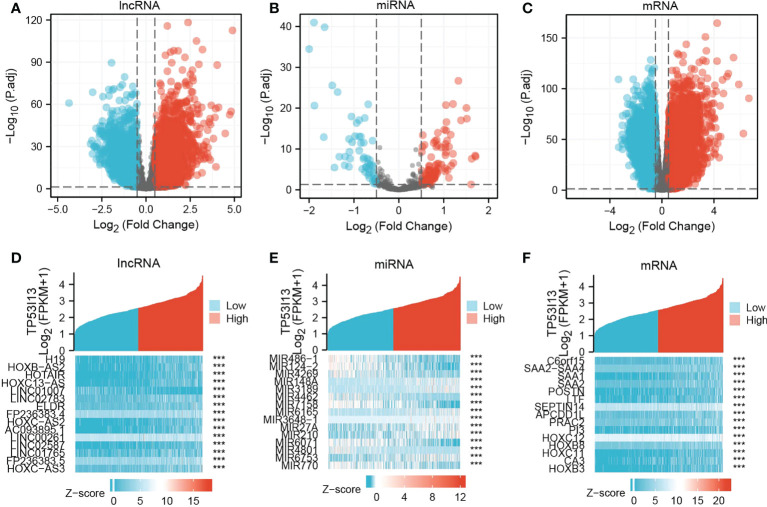
Volcano plots and heatmaps of DElncRNAs, DEmiRNAs, and DEmRNAs in glioma samples with low and high *TP53I13* expression levels. The volcano plots illustrate the **(A)** DElncRNAs, **(B)** DEmiRNAs, and **(C)** DEmRNAs. Heatmaps of 15 significant Differentially expressed genes (DEGs) closely correlated with **(D)** DElncRNAs, **(E)** DEmiRNAs, and **(F)** DEmRNAs.

Based on the above results, glioma patients’ outcomes can be predicted using *TP53I13* as a biomarker. In glioma samples, *TP53I13* expression appears to be lower than in paracancerous or cancerous tissues. Therefore, we confirmed this conjecture by identifying the DEmRNAs, DElncRNAs, and DEmiRNAs in glioma tissues with high and low TP53I13 expression and adjacent normal tissue using samples from the TCGA database. The threshold for lncRNA was set as |log fold change [FC]| > 0.5, and P < 0.05 was set as a threshold for the miRNA and mRNA. DElncRNA, DEmRNA, and DEmiRNA distribution was shown on a volcano plot (**Figures 6A-C**). There are 15 genes with differential expression in glioma and normal tissues with high and low levels of *TP53I13*, as shown by a heatmap. (**Figures 6D-F**).

### Molecular mechanism and biological function of *TP53I13*

In order to examine the molecular mechanism of *TP53I13* in gliomas as well as its relationship to other genes involved in glioma, the mRNA sequence data retrieved from TCGA database was analyzed using the functional module of LinkedOmics. The volcano plot reveals genes co-expressed with *TP53I13* using Pearson correlation (**Supplementary Figure 5A**). The genes negatively and positively linked to *TP5313* are highlighted by dark green and dark red dots, respectively. As shown in **Supplementary Figure 5B-C**, the heatmap shows the top 50 differentially expressed genes genes that negatively and positively correlated with *TP53I13* expression. STRING and GeneMANIA were used to investigate the function of the PPIs with *TP53I13* levels in gliomas. Based on PPI network analysis, the top 20 genes that tightly correlated with *TP53I13* were identified (**Supplementary Figure 5D**). Cytoscape was used to analyze the hub genes, and the results revealed a significant correlation between *TP53I13* and *TP53, TP53BP2, TP53I3, TP53INP1, GADD45B, UFL1*, and *PROSC* (**Supplementary Figure 5E**). The results show that these top genes were associated with transcriptional dysregulation in cancers. Further, the top 20 TP53I13-interacting proteins were identified using GeneMANIA software, and the proteins tightly interconnected with *AP1M2, AP1M1, TBX22, SPSB3, C19orf43, SMARCC22, FABP2, MAPK3, NAALADL1, FZD6*, and *SCRN2* (**Supplementary Figure 5F**).

**Figure 7:**
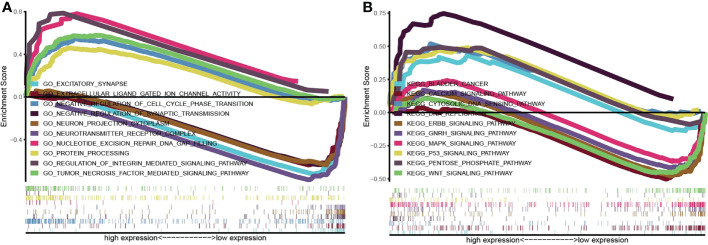
Gene set enrichment analysis was conducted to elucidate the biological function of TP53I13 in glioma. **(A)** Gene Ontology enrichment analysis. **(B)** Kyoto Encyclopedia of Genes and Genomes pathway enrichment analysis.

*TP53I13*’s biological function was analyzed using KEGG and GO pathways enriched in the TCGA dataset. The pathways enriched by *TP53I13* were body metabolism, negative regulation of excitatory synapse, cell cycle phase transition, negative regulation of synaptic transmission, regulation of integrin-mediated signaling pathway, protein processing, tumor necrosis factor-mediated signaling pathway, neurotransmitter receptor complex, nucleotide excision repair DNA gap filling, bladder cancer, cytosolic DNA sensing pathway, calcium signaling pathway, DNA replication, ERBB signaling pathway, GnRH signaling pathway, MAPK signaling pathway, p53 signaling pathway, pentose phosphate pathway, and WNT signaling pathway (**Figures 7A, B**).

### Analysis of *TP53I13* mutation in gliomas

**Figure 8:**
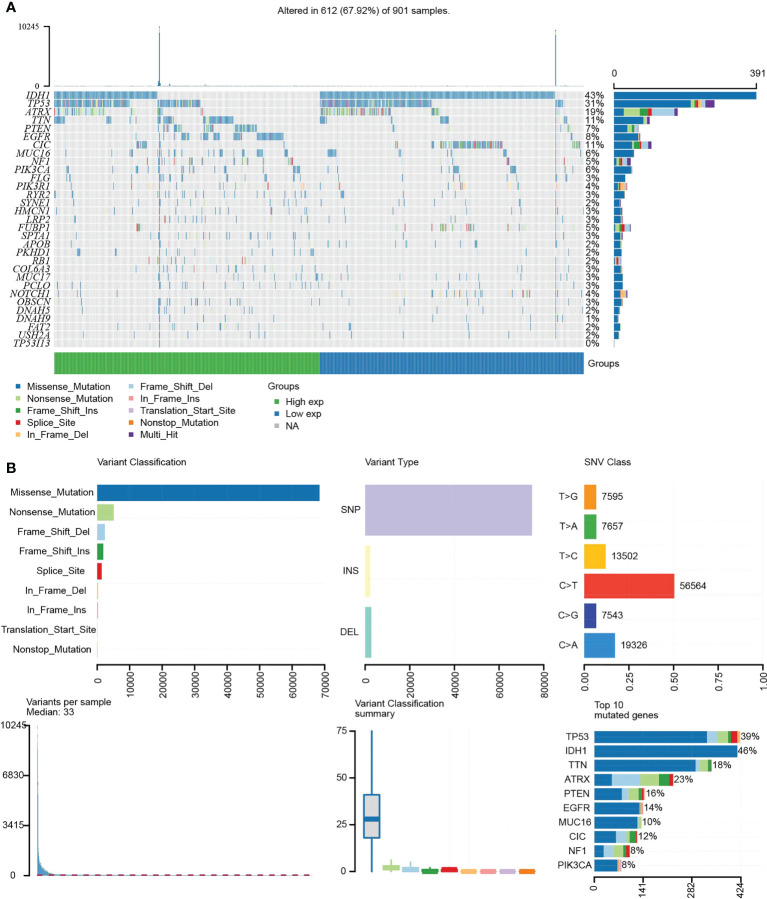
**(A)** Waterfall plot shows the mutation distribution of the top 30 commonly mutated genes. **(B)** The cohort summary diagram shows the variants distribution by variant type, classification, and SNV category. The bottom (left to right) showcases the mutation burden for each sample (variation classification type). The stacked bar chart reflects the top 10 mutated genes.

Tumor-specific mutations cause amino acid substitutions, which leads to mutated “neoantigens” and kill the tumor cells (21). A further examination of the relationship between *TP53I13* mutations and the TME was conducted based on the median expression of *TP53I13* between high- and low-immunity groups. In **Figure 8A**, the top 30 *TP53I13* expression genes with significant mutations are shown. *IDH1* was the top mutated gene in both low- and high-immunity groups and has been previously identified to be involved in tumorigenesis and cancer progression (22). Additionally, the study found that hyperimmune individuals showed more gene mutations than hypoimmune individuals, suggesting that glioma patients have more gene mutations, which are necessary for hyperimmune infiltration. An overview of the mutation profiles in glioma is shown in **Figure 8B**. As shown in **Supplementary Figure 6A**, we evaluated the connection between *TP53I13* expression and copy number variations (CNVs) using TIMER. The results show that in GBM, the infiltration of DC and CD4+ T cells were lower with chromosome arm-level gain of *TP53I13*, while in LGG, infiltration of B cells and DC were higher with chromosome arm-level deletion of *TP53I13*. Different immunological subgroups’ *TP53I13* expression was assessed, as seen in **Supplementary Figures 6B, C**. GBM cells expressed the highest level of *TP53I13*, while LGG cells expressed the highest level of *TP53I13*, indicating a better prognosis of glioma. Together these results suggest that *TP53I13* expression altered immune activity in the TME.

### Analysis of *TP53I13* expression in glioma at the single-cell level

For evaluating the link between the glioma patients and *TP53I13* expression at the single-cell level, scTIME and TISCH databases were analyzed. Data retrieved from the scTIME database showed that TP53I13 levels were higher in macrophages than in other cells except those with clonal mutations (**Supplementary Figure 6D**). Similar results were obtained from the TISCH database. As shown in **Supplementary Figure 6E**, high *TP53I13* expression was observed. Furthermore, *TP53I13* was shown to be expressed at higher levels in AC-like malignant cells and malignant cells according to the TSICH database, which further emphasized the malignancy of glioma severity and the necessity to find a biomarker for glioma treatment.

### Relationship between tumor immune infiltrating lymphocytes and *TP53I13* expression

**Figure 9:**
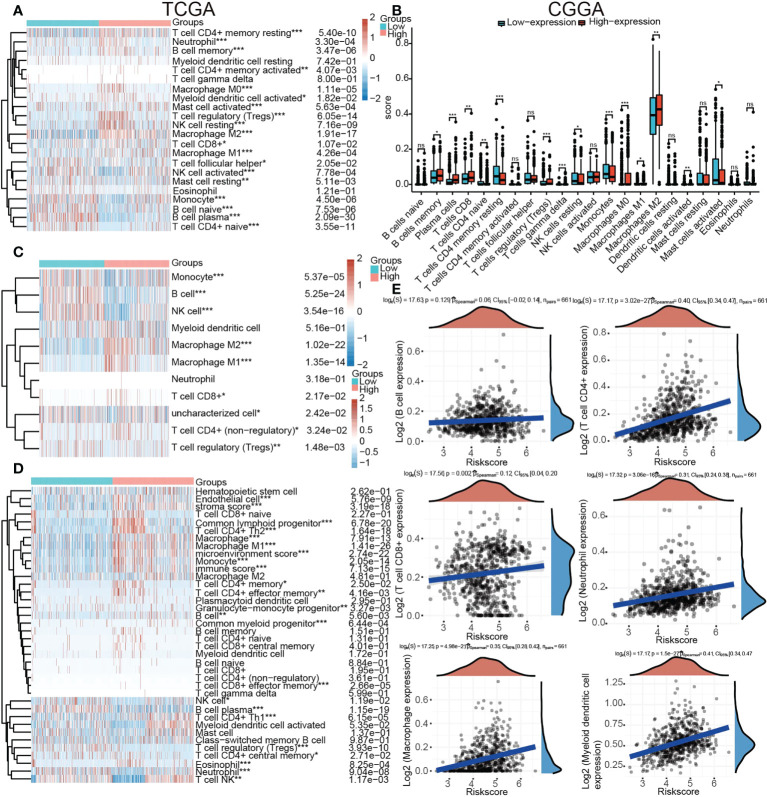
Relationships between *TP53I13* expression and different tumor immune lymphocytes **(A)** using the CIBERSORT algorithm on samples obtained from TCGA database, **(B)** using the CIBERSORT algorithm on samples obtained from CGGA database, **(C)** using the quanTiseq algorithm on samples obtained from on TCGA database, **(D)** by using the xCell algorithm on samples obtained from on TCGA database, and **(E)** by using the TIMER algorithm on samples obtained from on TCGA database. *P < 0.05, **P < 0.01, ***P < 0.001.

The proportion of 22 types of immune cells in glioma samples retrieved from CGGA and TCGA databases was sorted and analyzed by CIBERSORT to explore the relationship between *TP53I13* expression and tumor immune microenvironment. Analysis conducted on samples retrieved from the TCGA database revealed a significant increase in the proportion of neutrophils, resting memory CD4+ T cells, regulatory T-cells (Tregs), and M2 macrophages in the high-immunity group of glioma patients (**Figure 9A**). From the CGGA database, regulatory CD8+ T cells, memory B cells, macrophages, plasma cells, and T cells were found to be significantly increased. As a result of the treatment, resting NK cells, resting mast cells, and resting monocytes were all significantly reduced, as were naive CD4+ T cells, resting memory CD4+ T cells, and resting activated mast cells. (**Figure 9B**).

**Figure 10:**
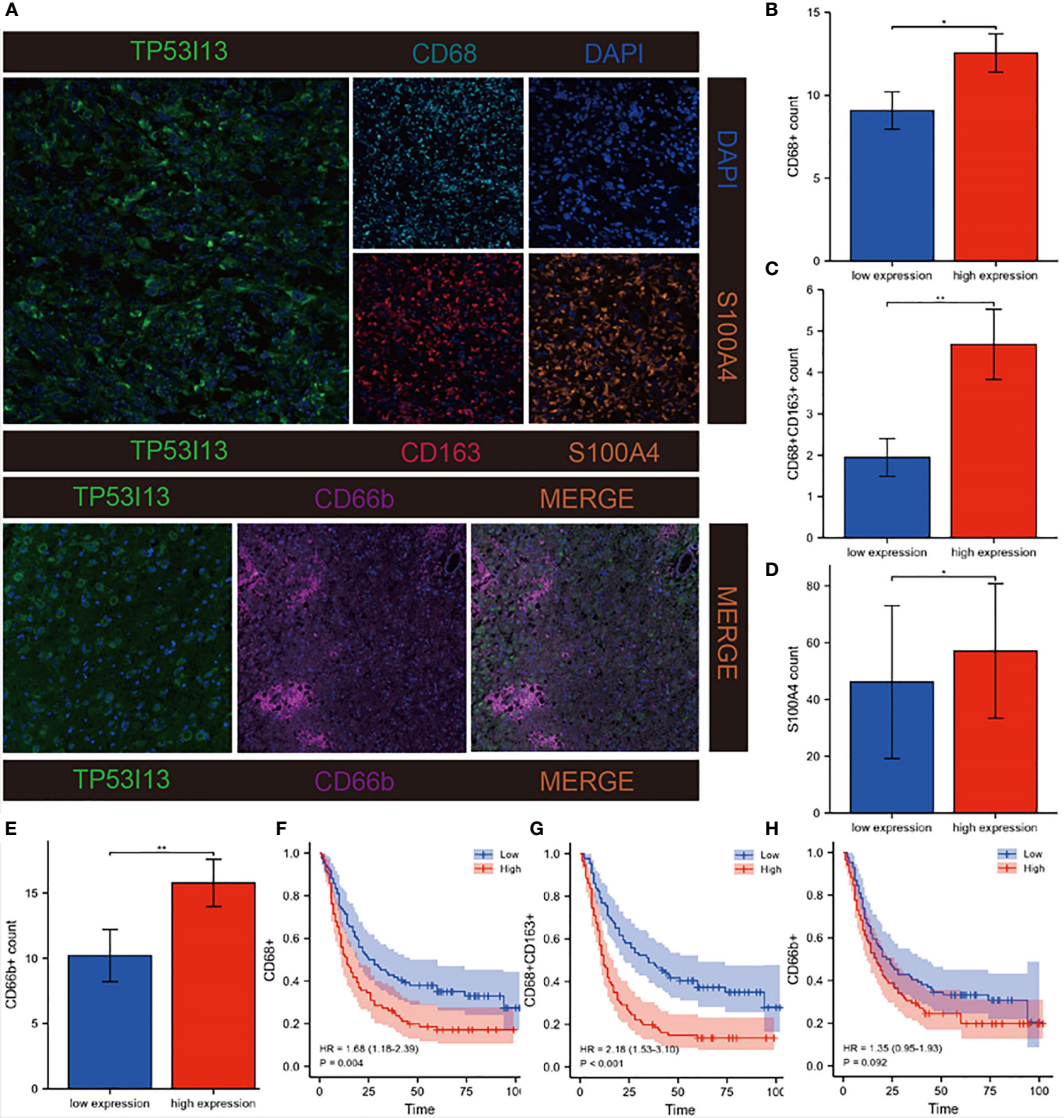
Investigation of correlations between *TP53I13* and macrophages, neutrophils, and CAFs markers on samples from Nantong University Affiliated Hospital. **(A)** mIHC of TP53I13 and different macrophage markers (CD68, CD163), neutrophils marker (CD66b), and CAFs marker (S100A4). **(B-E)** The relationships between the *TP53I13* expression and CD68+, CD68+CD163+, S100A4, and CD66b+. **(F-H)** Survival analysis of TP53I13 high- and low-expression and CD68+, CD68+CD163+, and CD66b+. *P < 0.05, **P < 0.01.

An algorithmic approach combining quanTiseq, xCell, and TIMER was employed to determine whether TP53I13 expression correlates with tumor immune lymphocytes. In glioma patients who had high levels of TP53I13 expression, the levels of M2 macrophages increased significantly, according to the quanTiseq algorithm (**Figure 9C**). The analysis performed using the xCell algorithm shows that the levels of M1 and M2 macrophages, common lymphoid progenitors, CD4+ Th_2_ cells, and neutrophils decreased in the low-immunity group compared to the high-immunity group (**Figure 9D**). Further, high-immunity groups showed significant growth in myeloid dendritic cells, neutrophils, macrophages, and CD4+ T cells based on the TIMER algorithm. (**Figure 9E**). The mIHC results confirm the relationship between *TP53I13* and macrophage markers such as (CD68 and CD163), neutrophils (CD66b), and fibroblasts (S100A4) (**Figure 10A**). Our results reveal a significant positive correlation between *TP53I13* expression and macrophages, neutrophils, and fibroblasts. Together, these analyses show that the high levels of macrophages, neutrophils, and fibroblasts in the high-immunity group facilitate tumor migration and development. By comparing the median expression levels of *TP53I13* in the samples, two groups were formed. According to the results, CD68+ expression in the low expression group of TP53I13 was lower than that in the high expression group of *TP53I13* (**Figure 10B**). Further, in the TP53I13 low expression group, compared with the *TP53I13* high expression group, the number of CD68+CD163+, S100A4, and CD68b+ cells decreased (**Figures 10C-E**). A high expression level of CD68+ and CD68+CD163+ led to a poor prognosis compared to a low expression level of CD68+ and CD68+CD163+ (**Figures 10F, G**). However, the prognostic value of CD66b+ was not remarkable (**Figure 10H**).

Further, we investigated the link between immune cell infiltration and the TP53I13 protein expression with radiotherapy status and tumor types in glioma patients using TIMER, CIBERSORT, quanTiseq, and the xCell algorithm. Regardless of the algorithm used, patients who underwent radiotherapy and expressed high TP53I13 levels had a high level of macrophages. It is interesting to note that radiotherapy has the lowest survival rate among patients with high expression levels of TP53I13, which may be associated with the increase in macrophage levels (**Figure 5**, **Supplementary Figure 7**).

According to a previous report, checkpoint blockade therapy induced immune cell infiltration in the TME (23). As a result, we explored different genes involved in immune checkpoints in relation to *TP53I13* (**Supplement Figure 8A**). Results showed that major immune checkpoint genes, such as CD44 (R = 0.51), LGALS9 (R = 0.51), LAIR1 (R = 0.51), CD274 (R = 0.39) and TNFRSF14 (R=0.69), are directly related to TP53I13 expression. Immunohistochemical localization shows that TP53I13 significantly correlates with CD274 (**Supplementary Figure 8B**).

Finally, the AUC values of Siglec15, CTLA-4, PD-L1 and *TP53I13* were compared, and the ROC curve was calculated to evaluate whether *TP53I13* could predict the immune infiltration of glioma. The results showed that *TP53I13* had higher predictive power than other markers (AUC = 0.822, 95%CI = 0.802-0.842) (**Supplementary Figures 9A-D**).

### Knockdown of *TP53I13* expression alters with cell migration and invasion, apoptosis, and cell cycle

**Figure 11:**
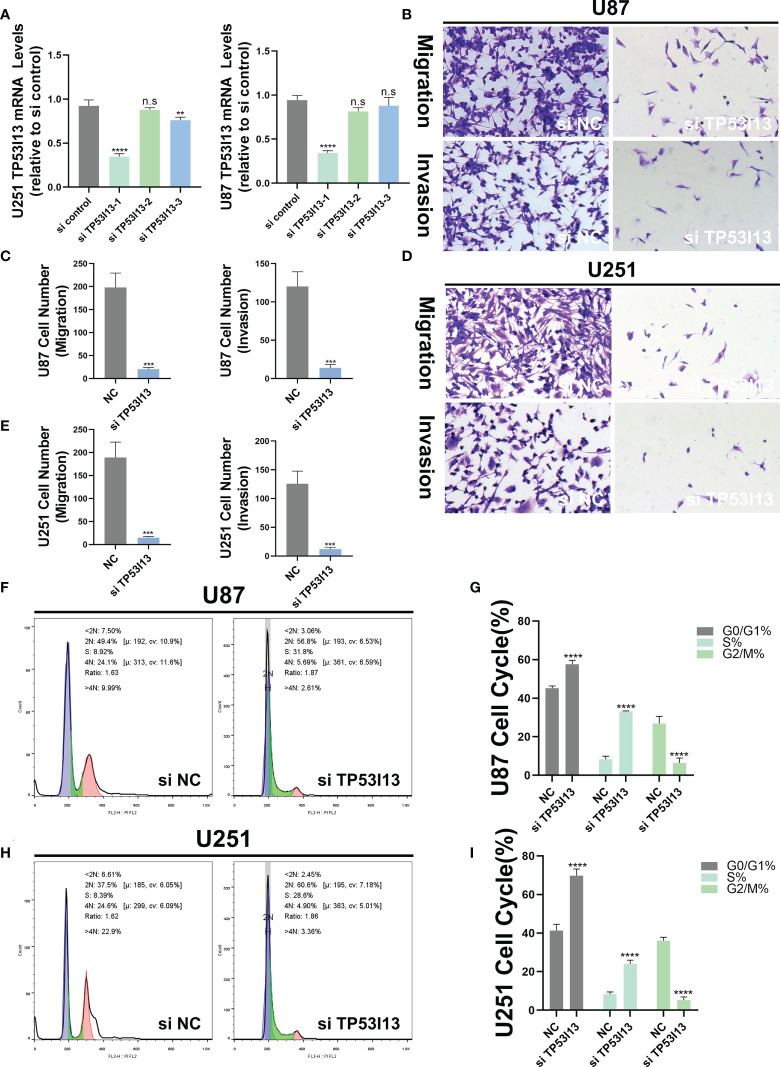
*TP53I13* promotes glioma cell migration and invasion *in vitro*. **(A)** Validation of siRNA interference efficiency in knockdown of *TP53I13* expression in U87 and U251 cells by RT-PCR **(B-E)** Transwell migration and invasion assay in the NC and *TP53I13* knockdown cells, and quantitative analysis of cell numbers. **(F-I)** Flow cytometry was performed on the NC and *TP53I13* knockdown cells to detect the cell cycle and to quantify the percentage of cells at different phases. **p ≤ 0.01, *** p ≤ 0.001 and ***p ≤ 0.0001. ns, no significance.

To investigate the biological function of *TP53I13*, the U87 and U251 cells were transfected with siRNA-NC and *TP53I13*-specific siRNAs (si-1, si-2, and si-3). qRT-PCR results reveal that siRNA1 was the most effective in silencing the expression of *TP53I13* after 48 h of treatment (**Figure 11A**). Cell migration and cell cycle were analyzed using the transwell assay and flow cytometry, respectively. TP53I13-siRNA1 transfected U87, and U251 cells showed diminished cell migration and invasion ability and increased apoptosis rate (**Figures 11B-E**). In cells knocked down for *TP53I13*, the percentage of S phase cells increased and the percentage of G2/M phase cells decreased (**Figures 11F-I**). Glioma cells with knockdown of TP53I13 expression exhibit reduced migration and invasion abilities and induce apoptosis.

## Discussion

Gliomas are classified as low- and high-grade by the World Health Organization (WHO). It is a lethal disease with a high CNV burden (24, 25). Moreover, it has been shown that focal lesions of the glioma (LGG or GBM) have a widespread influence, even in the hemisphere contralateral to the site of the lesion (26). The complicated pathogenesis of gliomas, the invasive behavior of this tumor, and the vigorous proliferative ability of the cells makes it challenging to treat gliomas (27, 28). Currently, few treatment choices are available for glioma patients, like surgery, radiation, and chemotherapy (29, 30). However, due to the low success rate, the outcomes of these therapies remain frustrating (31). Targeted immunotherapy is a novel treatment strategy for the treatment of glioma patients (32). Thus, identifying a new targeted therapeutic approach for treating glioma is the need of the hour.

When overexpressed, tumor protein p53 inducible protein 13 (TP53I13) plays a tumor suppressor role, thereby preventing tumor development. Genotoxic stressors, such as Adriamycin and/or UV irradiation, that increase the levels of *TP53I13* in a p53/TP53-dependent manner (33). In a previous study, downregulation of *TP53I13* was reported in adipose tissue in obese individuals, and its expression was reported in monocytes, macrophages, and adipocytes (34). In the presence of N4-Eru, elevated levels of *TP53I13* serve as a tumor suppressor in T-cell acute lymphoblastic leukemia (ALL) cells (Jurkat cells) (12). During the early stages of AD, the methylation levels of *TP53I13* are high (35). A significant role in the TME might be played by the upregulation of *TP53I13* expression in cancer and infiltrating immune cells.

In our study, data on glioma patients were retrieved from the CGGC and TCGA databases. It has been shown that *TP53I13* expression is higher in patients with gliomas. A significant correlation was found between *TP53I13* expression and tumor grade, chemotherapy, co-delete of 1p and 19q, and IDH mutations. These results indicate that a high *TP53I13* expression could be malignant to the cells. These results were further verified by immunohistochemistry and were consistent with bioinformatics analysis. Glioma patients’ *TP53I13* levels were directly associated with prognosis in Multivariate Cox analysis. *TP53I13* expression is also associated with a poor prognosis in glioma patients. Therefore, it would seem that *TP53I13* could be a potential therapeutic target and prognostic biomarker for gliomas. Further, the mechanism associated with *TP53I13* in glioma was investigated. It is likely that the levels of *TP53I13* expression may vary in tumor and paracancerous groups; hence, we explored this conjecture by analyzing DElncRNAs, DEmRNAs, and DEmiRNAs in glioma samples. Further, the top 50 genes which negatively and positively correlated with *TP53I13* were identified and analyzed to understand the molecular mechanisms associated with *TP53I13.* PPI analysis revealed that significant correlation between*TP53* and *TP53I13*. A previous study reported that *TP53* mutations and polymorphisms are frequently reported in glioma patients, which is the primary risk factor in gliomas (36). Studies have shown that knockdown of *TP53*-induced regulator of glycolysis and apoptosis (TIGAR) sensitizes glioma cells to hypoxia, irradiation, and temozolomide (37, 38). In mIDH1 mouse glioma model experiments, after TP53 and ATRX knockdown, glioma patients with *IDH1*-R132H exhibited increased DNA damage repair (DDR) activity and enhanced genomic stability (39). Therefore, a combination of DDR inhibitors and radiation might be an innovative therapeutic approach for treating glioma patients harboring *IDH1-*R132H mutation along with *ATRX* and *TP53* inactivating mutations (39). Studies have shown that mutations in *TP53* mutation are reported in 94% of glioma patients harboring IDH-mutation and patients without 1p/19q codeletion and is an important regulator of glioma progression (40, 41). Considering the close correlation between *TP53I13* and *TP53*, it is likely that *TP53I13* knockdown may increase sensitivity to radiation in glioma patients and decrease the progression of the disease. As a result of these results, *TP53I13* may become a potential biomarker for the treatment of gliomas in the future.

In order to determine how *TP53I13* functions biologically, GO and KEGG pathway enrichment analyses were performed. The results showed a correlation between*TP53I13* and different signaling pathways, including cell cycle, DNA replication, protein processing, and body metabolism. High *TP53I13* expression enriched pathways like ERBB, GNRH, MAPK, P53, and WNT signaling pathways, bladder cancer, tumor necrosis factor-mediated signaling pathway, embryonic development, and normal adult homeostasis (42). A study revealed that dysregulation of WNT signaling pathways is associated with the pathogenesis of various diseases (43). Therefore, high *TP53I13* expression resulting in poor survival outcomes may be associated with these pathways. In order to better understand *TP53I13*’s role in the pathogenesis of gliomas, *in vivo* models need to be validated. There is evidence that immune infiltration and tumor microenvironment play a crucial role in cancer pathogenesis (44, 45). Therefore, CIBERSORT, quanTiseq, xCell, and TIMER were used to investigate the correlation between TP53I13 expression and various tumor-infiltrating immune cells. The results show a positive correlation between *TP53I13* and macrophages. Macrophages are one of the most important immune cells and alter the tumor immune microenvironment by modulating the levels of angiogenic and immunosuppressive molecules (46, 47). Cytokines and chemokines secreted by macrophages are essential in regulating the immune response in complex tissue microenvironment (48). A study reports that glioma cells can activate macrophages, which further activates tumor cells (49). Additionally, macrophages account for 30–50% of the glioma TME and are found mainly in glioma cells (50). Further, macrophages aid in the growth of glioma cells, which could explain the increased malignancy and poor prognosis of high-grade glioma patients (51). In addition to macrophages, neutrophils also play a role in tumor metastasis (3). In addition, neutrophils express high Ki-67 levels, which is a marker for the degree of malignancy of the tumors. Using bioinformatics analysis and mIHC, we have identified a close relationship between *TP53I13* and neutrophils. Further *TP53I13* expression was higher in neutrophils, which suggests that *TP53I13* may promote tumor metastasis *via* neutrophils. Mounting evidence has shown that cancer-associated fibroblasts (CAFs) produce a variety of cytokines or metabolic products with immunogenic functions that can promote tumor invasion and metastasis. CAF can also alter the tumor matrix, which forms a barrier for drug or therapeutic immune cell infiltration, thereby preventing the influx of drugs and immune cells into the tumor tissue, which reduces the tumor therapeutic effect (52). S100A4 is a CAF marker; hence, the correlation between *TP53I13* and S100A4 was evaluated. The results show an increased *TP53I13* expression in CAF, elevated *TP53I13* expression in CAF may be associated with poor prognostic outcomes in glioma patients.

This study has enhanced the understanding of *TP53I13* expression in glioma patients. However, the study had several limitations. First, the sample size is one of the limitations of our study. The number of samples used was few; hence, additional samples are required to validate our findings further. Second, *TP53I13* functions and mechanisms in glioma need to be further explored.

Nevertheless, with a detailed bioinformatics analysis, we laid the groundwork for understanding *TP53I13*’s role in gliomas. Additionally, Nantong’s Affiliated Hospital provided 183 patient samples, confirming *TP53I13*’s prognostic value in predicting glioma outcomes. As a result, our research becomes even more valuable.

## Conclusion

Finally, high*TP53I13* expression was observed in glioma patients, resulting in poor prognosis and immune infiltration. In conclusion, our results suggest that *TP53I13* may serve as a potential diagnostic and treatment biomarker for glioma patients.

## Data availability statement

The datasets presented in this study can be found in online repositories. The names of the repository/repositories and accession number(s) can be found in the article/**Supplementary Material**.

## Ethics statement

The experimental protocol was established, according to the ethical guidelines of the Helsinki Declaration. Ethical permissions were granted by the Ethics Committee at Affiliated Hospital of Nantong University (No. 2018-K020). Written informed consent was obtained from individual or guardian participants. The patients/participants provided their written informed consent to participate in this study.

## Author contributions

XG, MX and TC performed most of the experiments, analyzed data, and wrote the manuscript. NH, PS and BL reviewed and edited the manuscript. ZW and JL is the guarantor of this work and has full access to all data in the study. The authors read and approved the final manuscript.

## Funding

This work was supported by the Scientific Research Project of Jiangsu Provincine Health Commission [grant number H2017052], China. Postgraduate Research & Practice Innovation Program of Jiangsu Province (No. SJCX21_1463).

## Acknowledgments

We acknowledge the Scientific Research Project of Jiangsu Provincine Health Commission [grant number H2017052] to support our study.

## Conflict of interest

The authors declare that the research was conducted in the absence of any commercial or financial relationships that could be construed as a potential conflict of interest.

## Publisher’s note

All claims expressed in this article are solely those of the authors and do not necessarily represent those of their affiliated organizations, or those of the publisher, the editors and the reviewers. Any product that may be evaluated in this article, or claim that may be made by its manufacturer, is not guaranteed or endorsed by the publisher.

## Supplementary material

Supplementary Figure 1
**(A)** Correlations between tumor and normal samples based on samples from Nantong University Affiliated Hospital. **(B)** Survival analysis of glioma patients having low and high expression levels of TP53I13 based on samples from Nantong University Affiliated Hospital. **(C)** Univariate Cox analysis of TP53I13 expression and clinical characteristics based on 159 glioma patients from Nantong University Affiliated Hospital.

Supplementary Figure 2 Boxplot shows relationships between TP53I13 expression and IDH mutation status based on **(A)** the CGGA mRNAseq_325 dataset, **(B)** the CGGA mRNAseq_693 dataset, and **(C)** the TCGA database. Boxplot shows relationships between TP53I13 expression and 1p/19q codeletion status based on **(D)** the CGGA mRNAseq_325 dataset, **(E)** the CGGA mRNAseq_693 dataset, and **(F)** the TCGA database.

Supplementary Figure 3 Comparison of Kaplan–Meier survival curves for OS of the low and high expression levels of TP53I13 in glioma based on clinical characteristics. **(A)** WHO grade 2, **(B)** WHO grade 3 and 4, **(C)** IDH wild-type, **(D)** IDH mutation, **(E)** 1p/19q codeletion, **(F)** 1p/19q non-codeletion, **(G)** Female, **(H)** Male.

Supplementary Figure 4 Comparison of Kaplan–Meier survival curves for DSS of TP53I13 high and low expression levels in glioma based on clinical characteristics. **(A)** WHO grade 2, **(B)** WHO grade 3 and 4, **(C)** *IDH* wildtype, **(D)** IDH mutation, **(E)** 1p/19q codeletion, **(F)** 1p/19q non-codeletion, **(G)** Female, **(H)** Male.

Supplementary Figure 5 Differentially expressed genes correlated with TP53I13 expression. **(A)** Relationships between TP53I13 and differentially expressed genes in glioma by using Pearson correlation. **(B)** Heatmaps illustrate the top 50 genes closely and positively correlated with TP53I13 in glioma. **(C)** Heatmaps show the top 50 genes closely and negatively correlated with TP53I13 in glioma. **(D)** PPI network for 20 key genes that are strongly correlated with TP53I13. **(E)** PPI network of TP53I13 analyzed by GeneMANIA. **(F)** Analysis of TP53I13-correlated proteins based on the PPI network by using the Cytoscape tool.

Supplementary Figure 6
**(A)** *TP53I13* copy number variation (CNV) influences infiltrating of neutrophils, dendritic cells, and CD4+ T cells in GBM and B cells and dendritic cells in LGG. The expression *TP53I13* in different immune cell subtypes in **(B)** GBM and **(C)** LGG. C1: wound healing; C2: IFN-gamma dominant; C3: inflammatory; C4: lymphocyte depleted; C5: immunologically quiet; C6: TGF-b dominant. Single-cell analysis of *TP53I13* expression in glioma based on the **(D)** scTIME database and **(E)** the TISCH database.

Supplementary Figure 7 Relationship between the high and low *TP53I13* expression and radiotherapy using **(A)** TIMER, **(B)** CIBERSORT, **(C)** quanTiseq, and **(D)** the xCell algorithm. G1: low *TP53I13* expression without radiotherapy; G2: low *TP53I13* expression with radiotherapy; G3: high *TP53I13* expression level without radiotherapy; G4: high *TP53I13* expression with radiotherapy.

Supplementary Figure 8
**(A)** Relationship between *TP53I13* and different immune checkpoint genes. **(B)** IHC was used to study the correlations between *TP53I13* and *CD274*

Supplementary Figure 9 Comparisons of predictive power for glioma infiltration between **(A)** *TP53I13* and common markers, such as **(B)** PD-L1, **(C)** CTLA-4, and **(D)** Siglec15.

Supplementary Table 4 The antibodies used in this study.
