# Supplementary figures and images for: TP53I13 promotes metastasis in glioma via macrophages, neutrophils, and fibroblasts and is a potential prognostic biomarker
Source: Front Immunol. 2022 Oct 7;13:974346. doi: 10.3389/fimmu.2022.974346 (PMC9585303; doi:10.3389/fimmu.2022.974346)

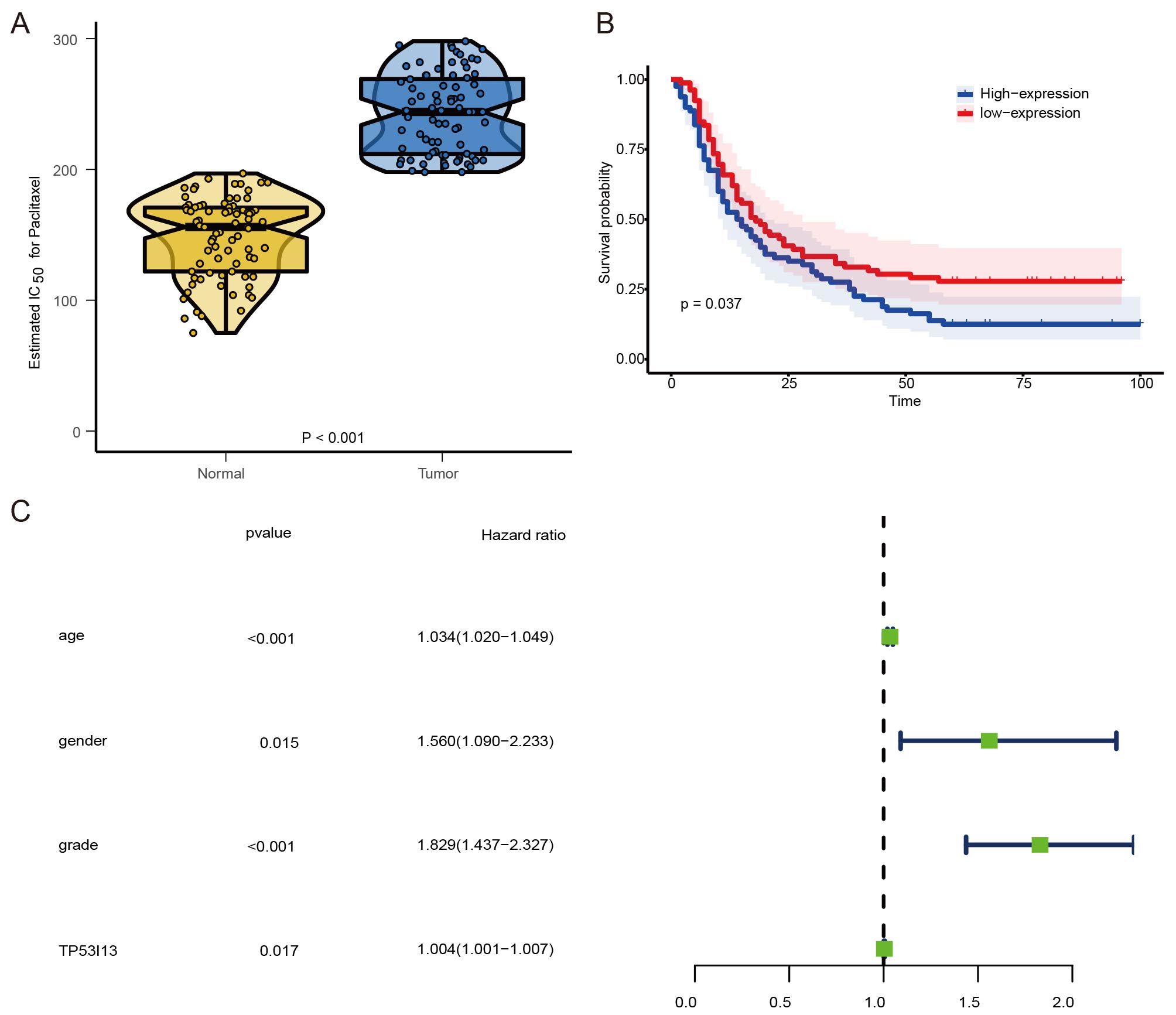

Supplement: Supplementary Figure 1 — (A) Correlations between tumor and normal samples based on samples from Nantong University Affiliated Hospital. (B) Survival analysis of glioma patients having low and high expression levels of TP53I13 based on samples from Nantong University Affiliated Hospital. (C) Univariate Cox analysis of TP53I13 expression and clinical characteristics based on 159 glioma patients from Nantong University Affiliated Hospital. [file Image_1.jpeg]

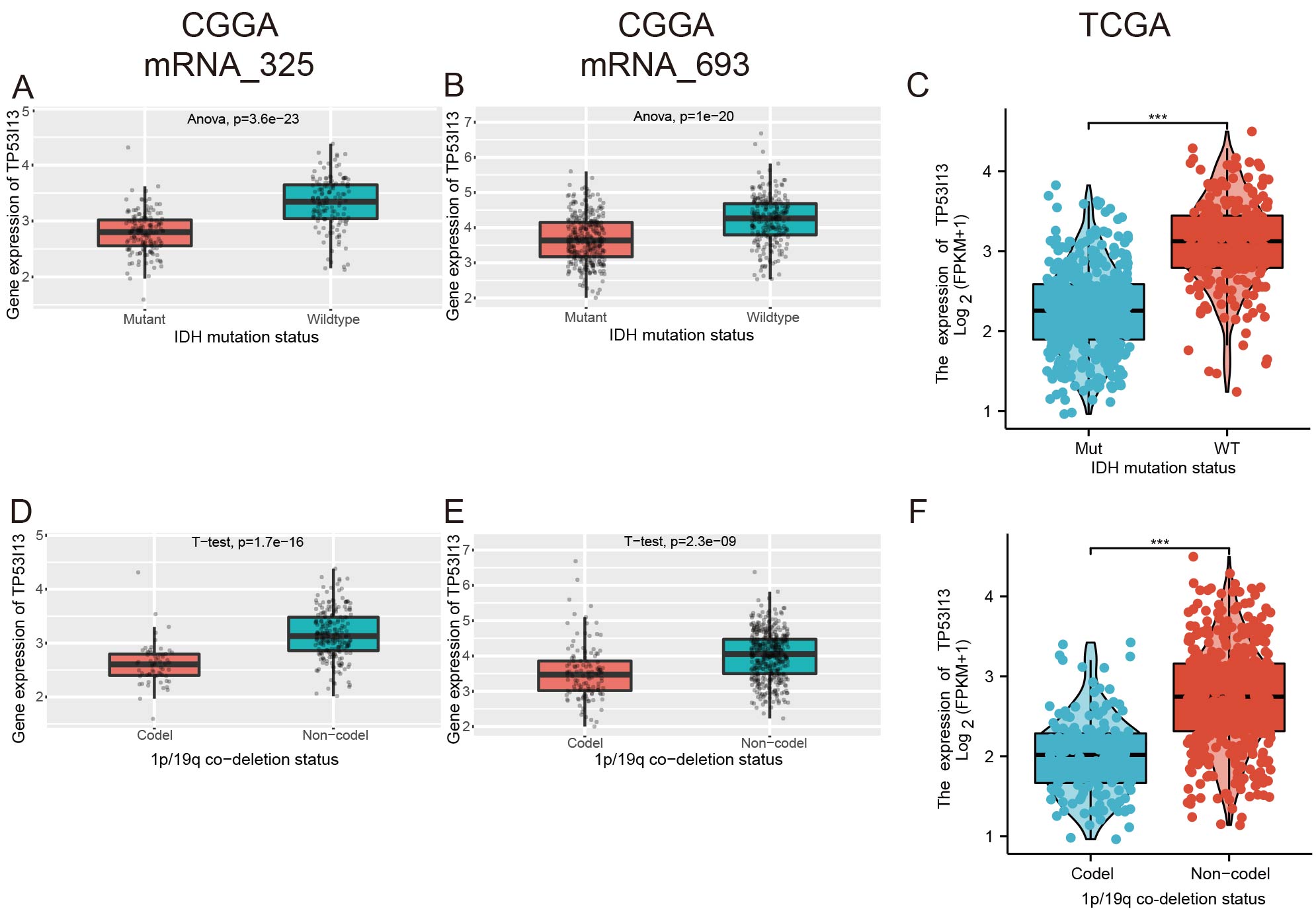

Supplement: Supplementary Figure 2 — Boxplot shows relationships between TP53I13 expression and IDH mutation status based on (A) the CGGA mRNAseq_325 dataset, (B) the CGGA mRNAseq_693 dataset, and (C) the TCGA database. Boxplot shows relationships between TP53I13 expression and 1p/19q codeletion status based on (D) the CGGA mRNAseq_325 dataset, (E) the CGGA mRNAseq_693 dataset, and (F) the TCGA database. [file Image_2.jpeg]

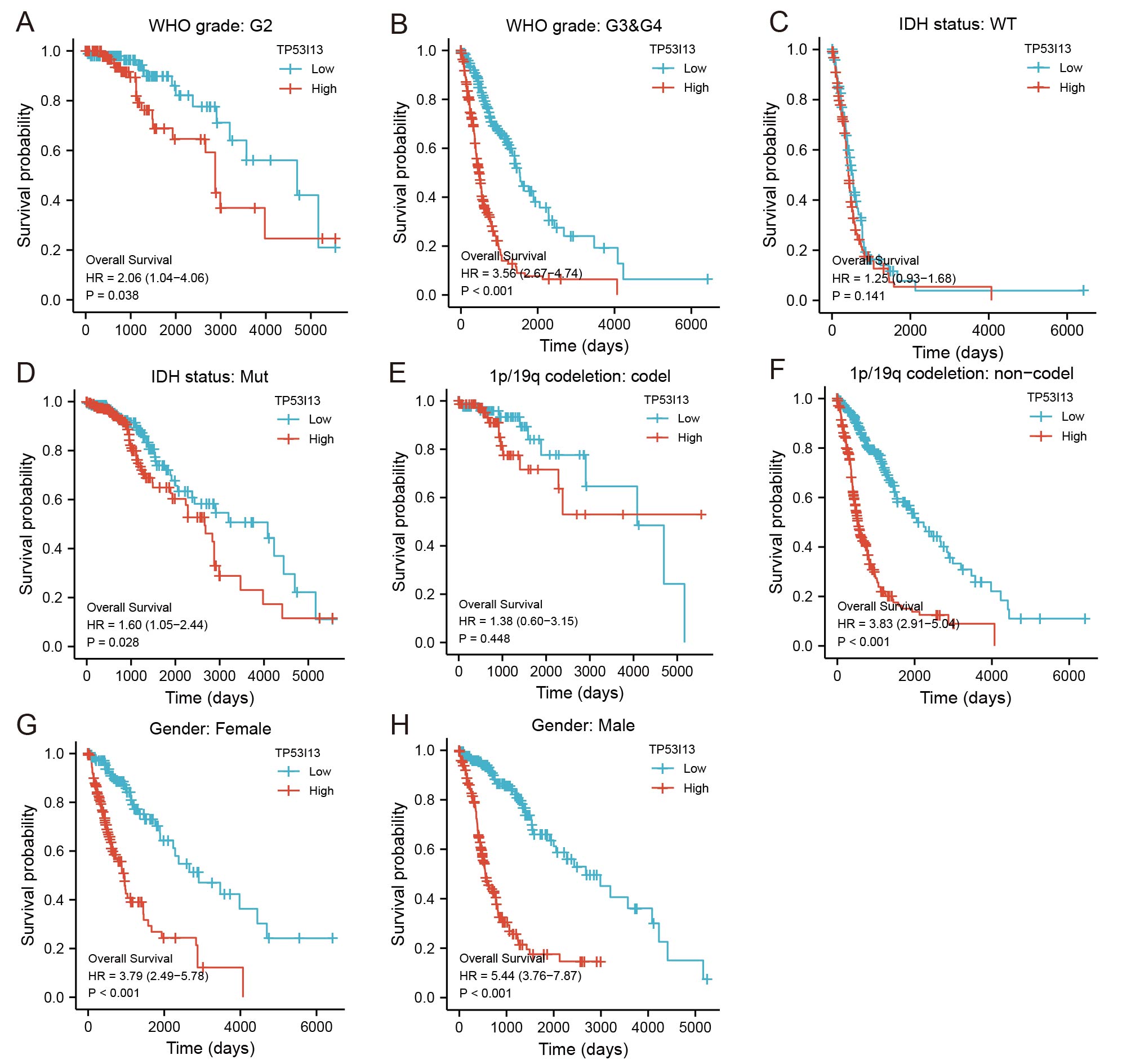

Supplement: Supplementary Figure 3 — Comparison of Kaplan–Meier survival curves for OS of the low and high expression levels of TP53I13 in glioma based on clinical characteristics. (A) WHO grade 2, (B) WHO grade 3 and 4, (C) IDH wild-type, (D) IDH mutation, (E) 1p/19q codeletion, (F) 1p/19q non-codeletion, (G) Female, (H) Male. [file Image_3.jpeg]

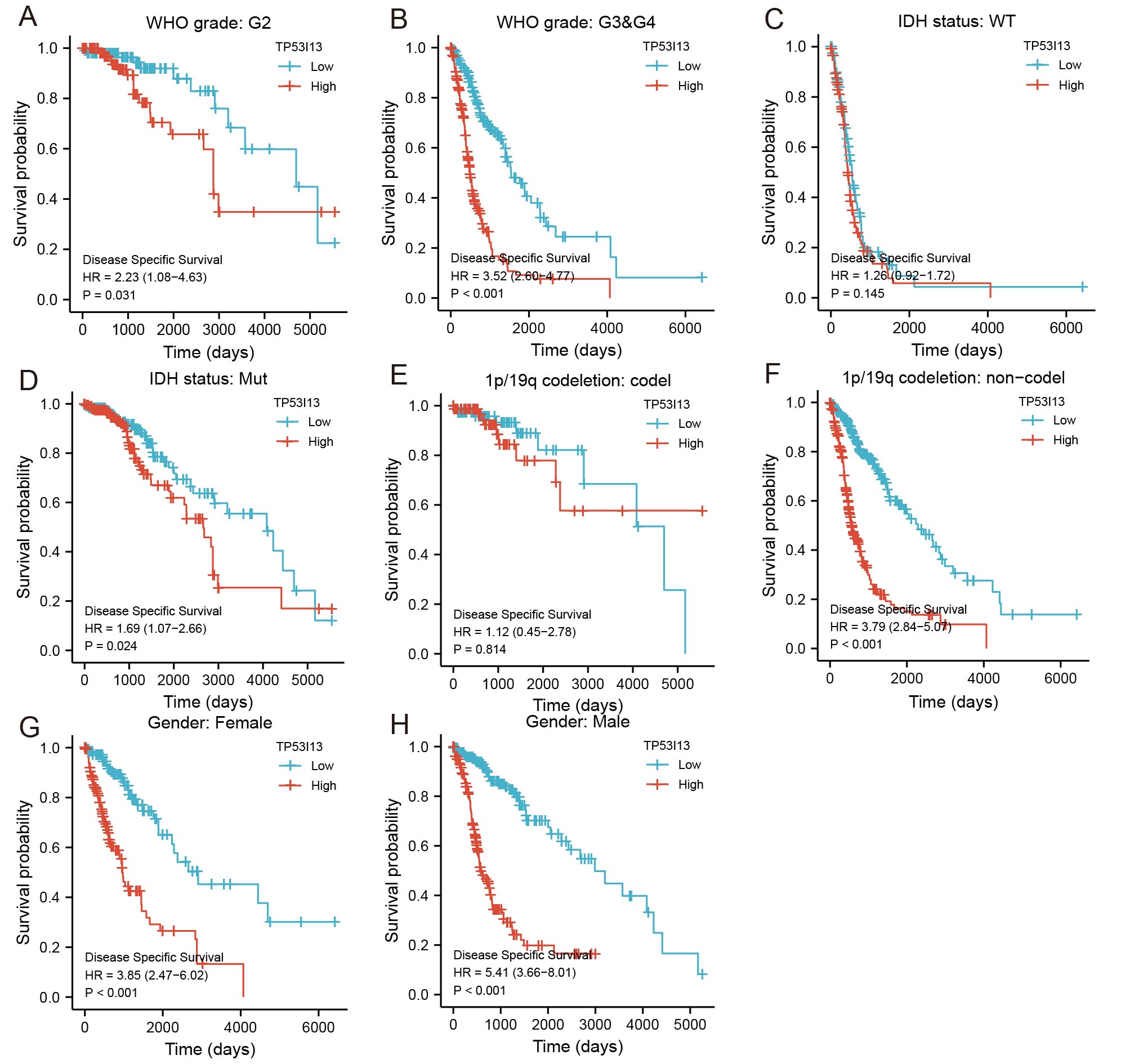

Supplement: Supplementary Figure 4 — Comparison of Kaplan–Meier survival curves for DSS of TP53I13 high and low expression levels in glioma based on clinical characteristics. (A) WHO grade 2, (B) WHO grade 3 and 4, (C) IDH wildtype, (D) IDH mutation, (E) 1p/19q codeletion, (F) 1p/19q non-codeletion, (G) Female, (H) Male. [file Image_4.jpeg]

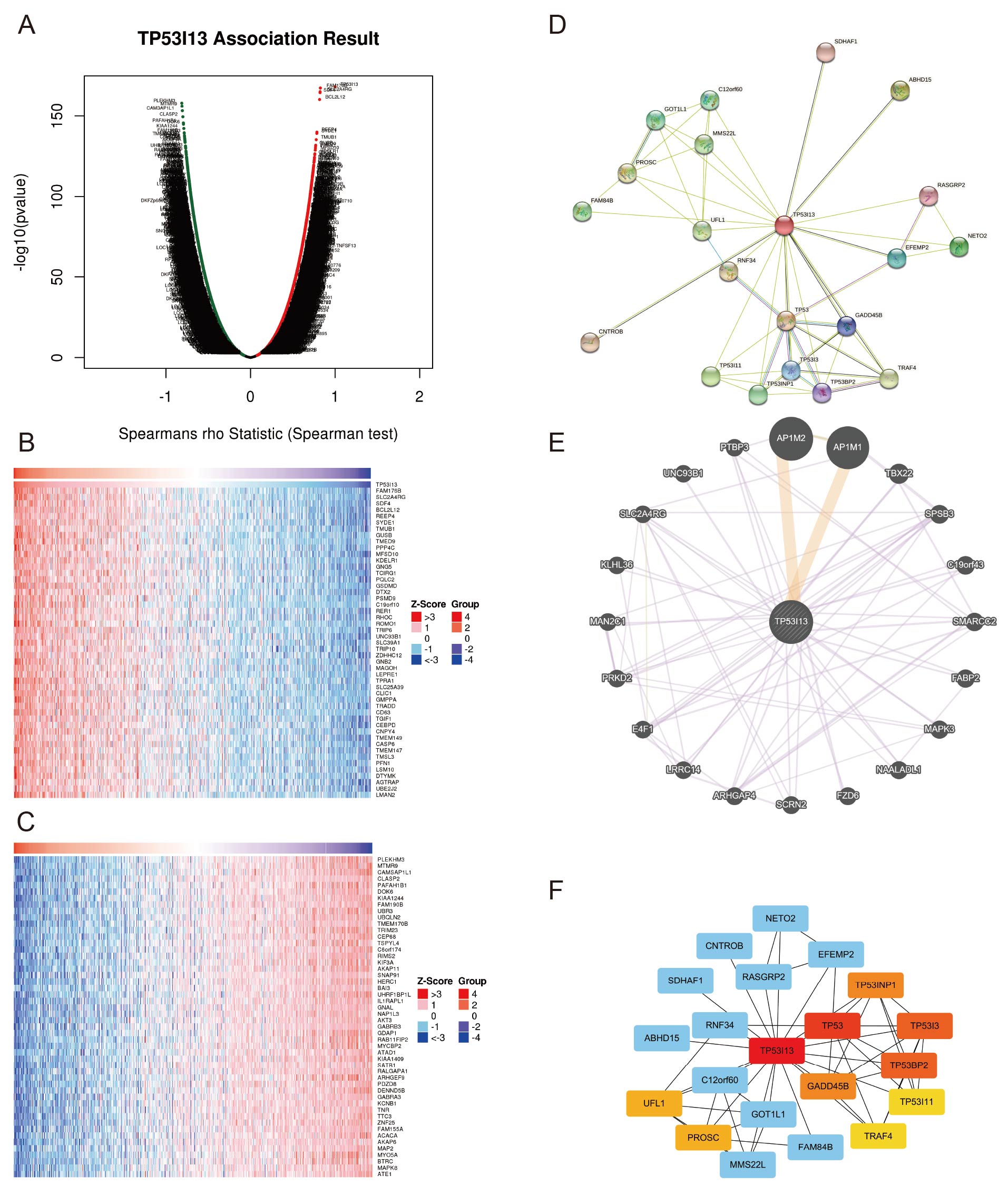

Supplement: Supplementary Figure 5 — Differentially expressed genes correlated with TP53I13 expression. (A) Relationships between TP53I13 and differentially expressed genes in glioma by using Pearson correlation. (B) Heatmaps illustrate the top 50 genes closely and positively correlated with TP53I13 in glioma. (C) Heatmaps show the top 50 genes closely and negatively correlated with TP53I13 in glioma. (D) PPI network for 20 key genes that are strongly correlated with TP53I13. (E) PPI network of TP53I13 analyzed by GeneMANIA. (F) Analysis of TP53I13-correlated proteins based on the PPI network by using the Cytoscape tool. [file Image_5.jpeg]

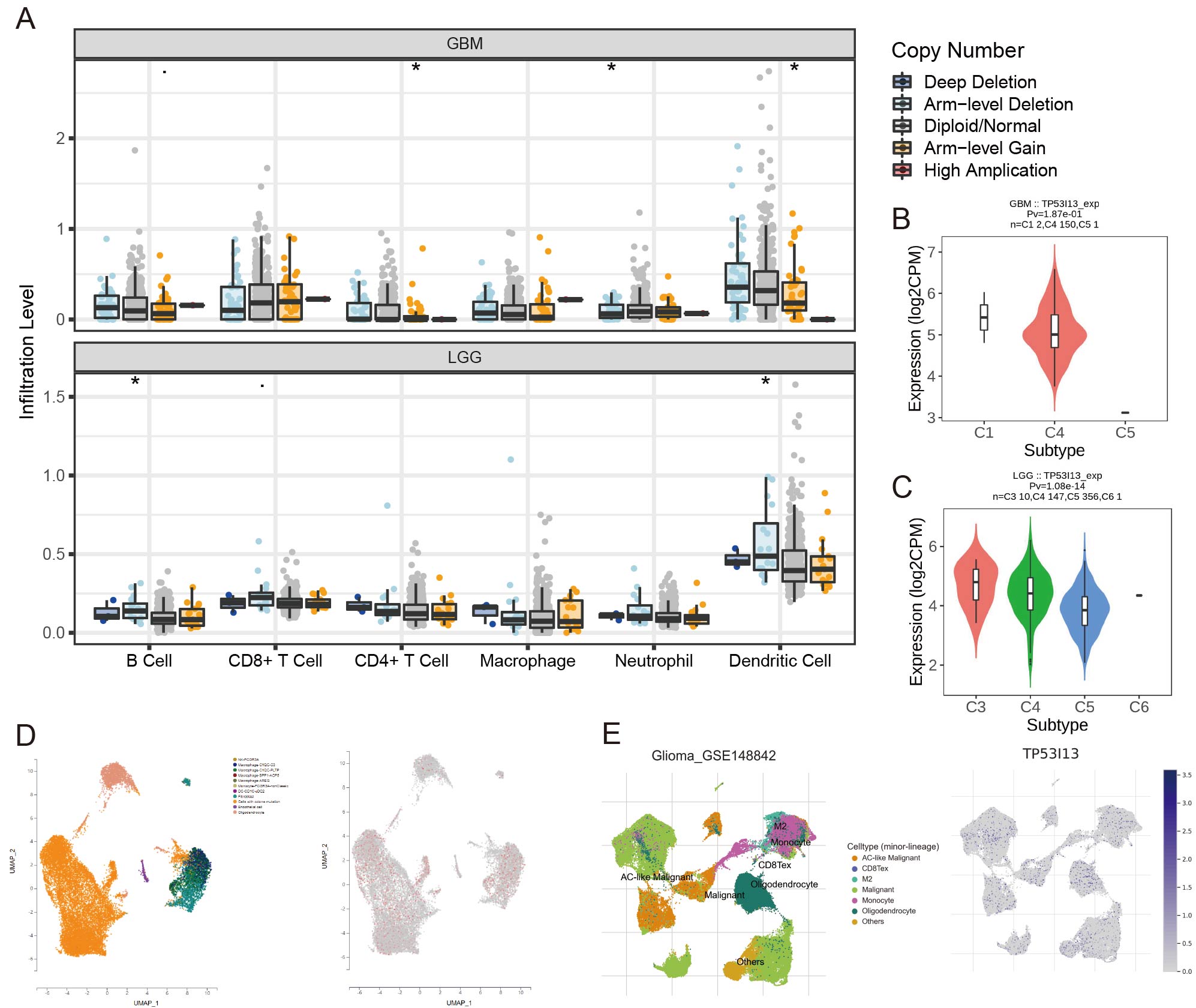

Supplement: Supplementary Figure 6 — (A) TP53I13 copy number variation (CNV) influences infiltrating of neutrophils, dendritic cells, and CD4+ T cells in GBM and B cells and dendritic cells in LGG. The expression TP53I13 in different immune cell subtypes in (B) GBM and (C) LGG. C1: wound healing; C2: IFN-gamma dominant; C3: inflammatory; C4: lymphocyte depleted; C5: immunologically quiet; C6: TGF-b dominant. Single-cell analysis of TP53I13 expression in glioma based on the (D) scTIME database and (E) the TISCH database. [file Image_6.jpeg]

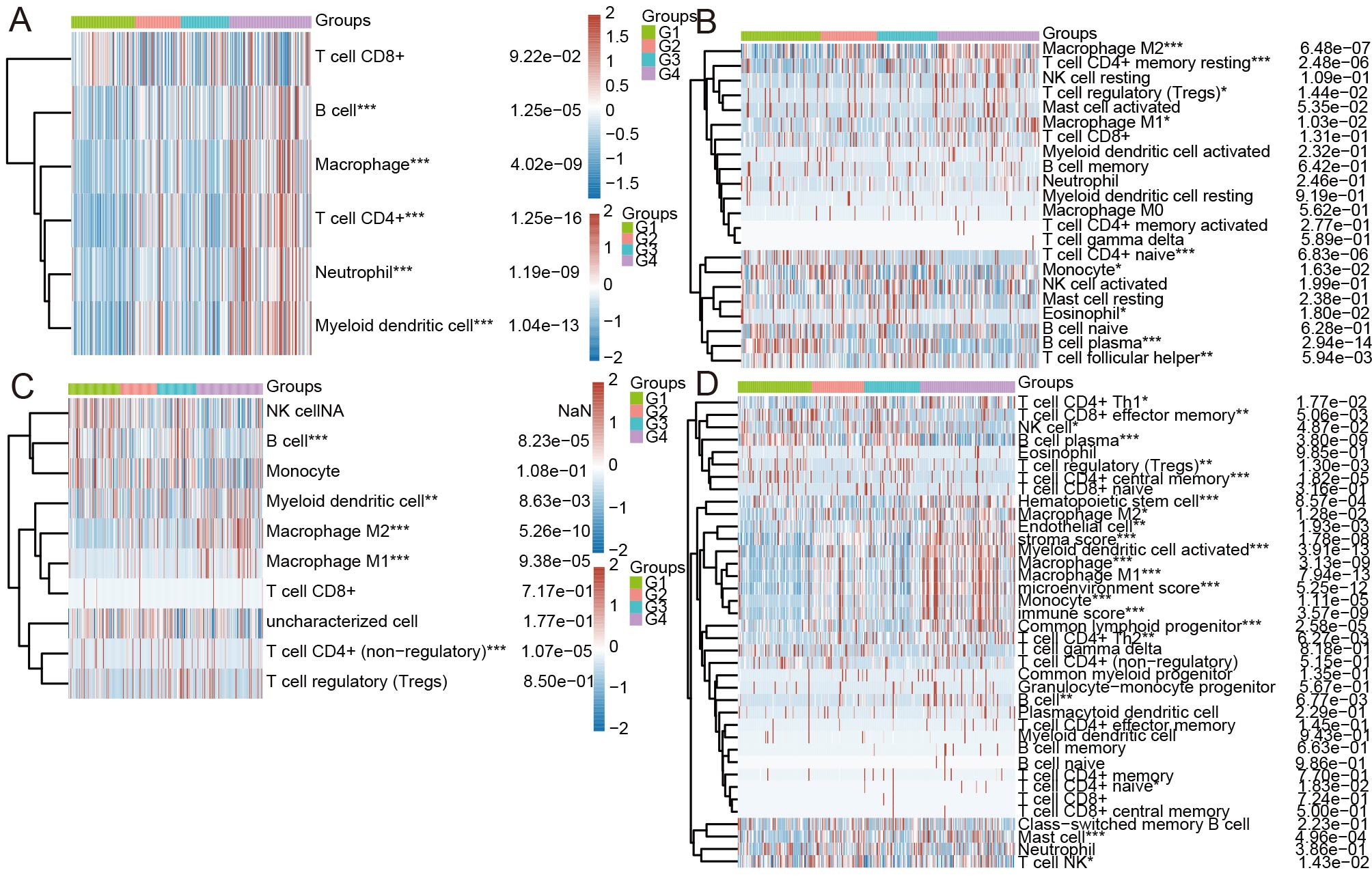

Supplement: Supplementary Figure 7 — Relationship between the high and low TP53I13 expression and radiotherapy using (A) TIMER, (B) CIBERSORT, (C) quanTiseq, and (D) the xCell algorithm. G1: low TP53I13 expression without radiotherapy; G2: low TP53I13 expression with radiotherapy; G3: high TP53I13 expression level without radiotherapy; G4: high TP53I13 expression with radiotherapy. [file Image_7.jpeg]

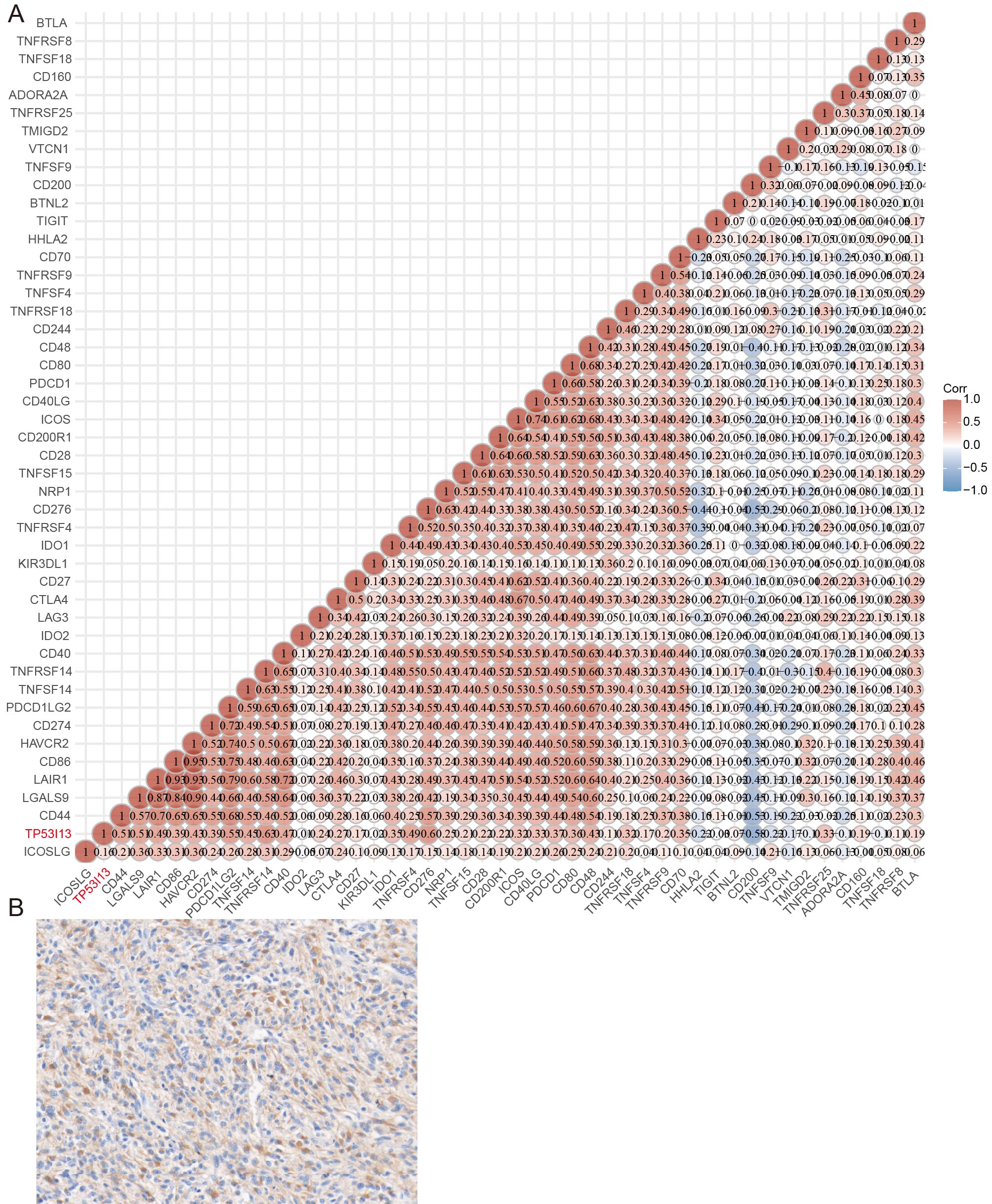

Supplement: Supplementary Figure 8 — (A) Relationship between TP53I13 and different immune checkpoint genes. (B) IHC was used to study the correlations between TP53I13 and CD274 [file Image_8.jpeg]

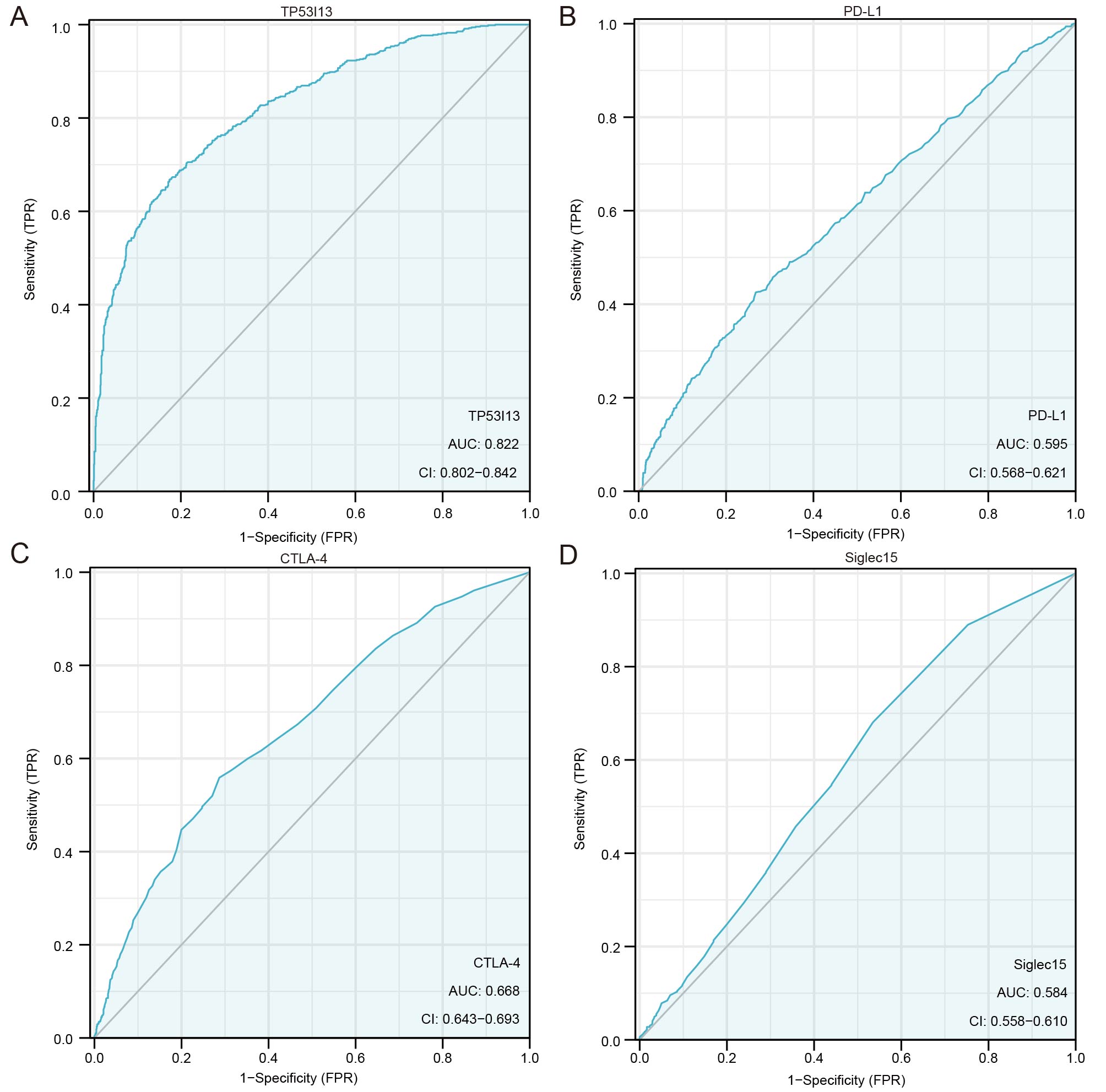

Supplement: Supplementary Figure 9 — Comparisons of predictive power for glioma infiltration between (A) TP53I13 and common markers, such as (B) PD-L1, (C) CTLA-4, and (D) Siglec15. [file Image_9.jpeg]
