# Supplementary material for: TP53I13 promotes metastasis in glioma via macrophages, neutrophils, and fibroblasts and is a potential prognostic biomarker
Source: Front Immunol. 2022 Oct 7;13:974346. doi: 10.3389/fimmu.2022.974346 (PMC9585303; doi:10.3389/fimmu.2022.974346)
Supplement: Supplementary Table 4 — The antibodies used in this study. [file Table_4.docx]

| **Antibody** | **Source** | **Identifier** | **Dilution** |
| --- | --- | --- | --- |
| rabbit anti-human CD68 antibody | Cell Signaling Technology | D4B9C | 1:1500 |
| rabbit anti-human S100A4 antibody | Abcam | ab197896 | 1:250 |
| rabbit anti-human CD163 antibody | Cell Signaling Technology | D6U1J | 1:2000 |
| rabbit anti-human PDL1 antibody | Cell Signaling Technology | 13684S | 1:20 |
| mouse anti-human Arigo CD66b antibody | Agrio | arg66287 | 1:500 |
