# Supplementary material for: TP53I13 promotes metastasis in glioma via macrophages, neutrophils, and fibroblasts and is a potential prognostic biomarker
Source: Front Immunol. 2022 Oct 7;13:974346. doi: 10.3389/fimmu.2022.974346 (PMC9585303; doi:10.3389/fimmu.2022.974346)
Supplement: Supplementary file 11 [file Table_1.docx]

Supplementary Table 1

The clinical samples collected from Affiliated Hospital of Nantong University

| **Tissue microarray chip1 (24 glioma samples)** | | | | | | | |
| --- | --- | --- | --- | --- | --- | --- | --- |
| **Patient ID** | **Gender** | **Age (years)** | **Anatomic Site** | **Grade** | **Death** | **Month** | **IHC-score of TP53I13** |
| 1 | female | 49 | Right temporal lobe | Grade4 | 1 | 15 | 235 |
| 2 | female | 51 | Right frontotemporal lobe | Grade2 | 1 | 11 | 263 |
| 3 | male | 61 | Temporal parietal lobe | Grade4 | 1 | 1 | 247 |
| 4 | female | 42 | Left temporal lobe | Grade3 | 1 | 18 | 167 |
| 5 | male | 53 | Right frontal lobe | Grade3 | 1 | 12 | 252 |
| 6 | female | 51 | Left frontal lobe | Grade2 | 0 | 96 | 189 |
| 7 | male | 43 | Top right | Grade3 | 0 | 60 | 207 |
| 8 | male | 49 | Right basal ganglia | Grade2 | 1 | 6 | 244 |
| 9 | male | 64 | Right occipital part | Grade3 | 1 | 6 | 167 |
| 10 | female | 46 | Left temporal lobe | Grade3 | 0 | 60 | 254 |
| 11 | male | 65 | Right occipital part | Grade2 | 0 | 100 | 231 |
| 12 | female | 25 | Left temporal basal ganglia | Grade2 | 0 | 92 | 140 |
| 13 | male | 47 | Bifrontal part | Grade3 | 1 | 19 | 257 |
| 14 | male | 49 | Head | Grade2 | 1 | 28 | 173 |
| 15 | female | 67 | Left temporal lobe | Grade4 | 1 | 6 | 190 |
| 16 | male | 34 | Cerebellum | Grade4 | 0 | 86 | 166 |
| 17 | male | 42 | Head | Grade3 | 1 | 17 | 221 |
| 18 | male | 64 | Right temporal parietal | Grade3 | 1 | 20 | 151 |
| 19 | male | 33 | Left temporal lobe | Grade3 | 0 | 84 | 178 |
| 20 | male | 39 | Left frontal part | Grade2 | 0 | 86 | 112 |
| 21 | male | 47 | Right temporal lobe | Grade4 | 1 | 2 | 240 |
| 22 | male | 65 | Right occipital | Grade3 | 1 | 32 | 211 |
| 23 | male | 57 | Left frontotemporal | Grade3 | 1 | 2 | 168 |
| 24 | female | 63 | Frontal lobe | Grade3 | 1 | 41 | 211 |
| **Tissue microarray chip2 (25 glioma samples)** | | | | | | | |
| **Patient ID** | **Gender** | **Age (years)** | **Anatomic Site** | **Grade** | **Death** | **Month** | **IHC-score of TP53I13** |
| 1 | male | 47 | Right parietal lobe | Grade3 | 1 | 9 | 118 |
| 2 | male | 71 | Right frontal lobe | Grade4 | 1 | 17 | 122 |
| 3 | male | 57 | Head | Grade2 | 1 | 8 | 75 |
| 4 | male | 65 | Left occipital | Grade3 | 1 | 8 | 128 |
| 5 | male | 73 | Right temporal | Grade3 | 1 | 11 | 88 |
| 6 | male | 62 | Left temporal lobe | Grade3 | 1 | 5 | 111 |
| 7 | male | 63 | Left frontal lobe | Grade4 | 1 | 7 | 132 |
| 8 | male | 64 | Left frontal lobe | Grade2 | 0 | 60 | 149 |
| 9 | female | 62 | Left frontal lobe | Grade2 | 0 | 81 | 156 |
| 10 | male | 56 | Head | Grade2 | 1 | 8 | 214 |
| 11 | male | 71 | Left temporal lobe | Grade4 | 1 | 17 | 172 |
| 12 | female | 49 | Temporal lobe | Grade3 | 0 | 60 | 92 |
| 13 | male | 60 | Right frontal lobe | Grade3 | 1 | 9 | 184 |
| 14 | female | 50 | Left frontal lobe | Grade2 | 0 | 79 | 198 |
| 15 | female | 46 | Left temporal lobe | Grade3 | 0 | 78 | 147 |
| 16 | female | 61 | Left frontotemporal lobe | Grade4 | 1 | 7 | 203 |
| 17 | male | 49 | Right frontal lobe | Grade2 | 0 | 77 | 91 |
| 18 | female | 36 | Left occipital part | Grade2 | 0 | 77 | 133 |
| 19 | male | 58 | Fourth ventricle | Grade3 | 1 | 6 | 171 |
| 20 | male | 50 | Temporal lobe of brain | Grade3 | 1 | 5 | 199 |
| 21 | male | 60 | Right frontal lobe | Grade2 | 0 | 76 | 145 |
| 22 | male | 73 | Right frontotemporal lobe | Grade4 | 1 | 5 | 202 |
| 23 | female | 40 | Left frontal lobe | Grade2 | 0 | 76 | 104 |
| 24 | female | 42 | Left temporal lobe | Grade3 | 0 | 76 | 169 |
| 25 | male | 40 | Right frontotemporal lobe | Grade2 | 1 | 14 | 193 |
| **Tissue microarray chip3 (29 glioma samples)** | | | | | | | |
| **Patient ID** | **Gender** | **Age (years)** | **Anatomic Site** | **Grade** | **Death** | **Month** | **IHC-score of TP53I13** |
| 1 | female | 44 | Right frontal lobe | Grade4 | 1 | 25 | 230 |
| 2 | male | 44 | Right frontotemporal lobe and basal ganglia | Grade3 | 1 | 14 | 212 |
| 3 | male | 38 | Right frontal lobe | Grade3 | 0 | 61 | 155 |
| 4 | female | 76 | Left frontal | Grade4 | 1 | 17 | 295 |
| 5 | female | 58 | Left frontal lobe | Grade3 | 1 | 14 | 245 |
| 6 | female | 49 | Right parietal lobe | Grade2 | 0 | 60 | 161 |
| 7 | female | 33 | Right frontal lobe | Grade3 | 0 | 63 | 204 |
| 8 | female | 27 | Left temporal fundus | Grade4 | 0 | 63 | 290 |
| 9 | male | 66 | Right brain | Grade4 | 1 | 16 | 254 |
| 10 | female | 59 | Head | Grade4 | 1 | 11 | 270 |
| 11 | male | 73 | Right temporal | Grade4 | 1 | 6 | 189 |
| 12 | female | 62 | Right frontotemporal | Grade4 | 1 | 22 | 264 |
| 13 | female | 57 | Intracranial | Grade4 | 1 | 6 | 282 |
| 14 | female | 61 | Left frontal lobe | Grade3 | 0 | 65 | 162 |
| 15 | female | 68 | Left frontotemporal parietal | Grade4 | 1 | 3 | 273 |
| 16 | male | 65 | Temporal floor | Grade4 | 1 | 28 | 292 |
| 17 | female | 44 | Left frontal lobe | Grade4 | 0 | 67 | 252 |
| 18 | female | 63 | frontal lobe | Grade4 | 1 | 6 | 269 |
| 19 | male | 43 | Right temporal | Grade3 | 1 | 51 | 207 |
| 20 | male | 56 | Right temporal occipital region | Grade4 | 1 | 20 | 285 |
| 21 | male | 61 | Right temporal | Grade4 | 1 | 10 | 288 |
| 22 | female | 67 | Left temporal | Grade4 | 0 | 68 | 262 |
| 23 | female | 39 | Head | Grade4 | 1 | 1 | 282 |
| 24 | female | 57 | Left frontotemporal lobe | Grade4 | 1 | 3 | 278 |
| 25 | male | 54 | Right frontal lobe | Grade2 | 1 | 13 | 197 |
| 26 | female | 68 | Left frontal lobe | Grade4 | 1 | 38 | 272 |
| 27 | female | 59 | Right frontal lobe | Grade4 | 1 | 7 | 279 |
| 28 | female | 49 | Right frontal lobe | Grade2 | 1 | 45 | 210 |
| 29 | male | 65 | Right frontotemporal lobe | Grade3 | 1 | 6 | 235 |
| **Tissue microarray chip4 (23 glioma samples)** | | | | | | | |
| **Patient ID** | **Gender** | **Age (years)** | **Anatomic Site** | **Grade** | **Death** | **Month** | **IHC-score of TP53I13** |
| 1 | female | 44 | Head | Grade2 | 0 | 60 | 110 |
| 2 | male | 47 | Right top pillow | Grade4 | 1 | 17 | 102 |
| 3 | male | 47 | Right temporal lobe | Grade3 | 1 | 10 | 238 |
| 4 | male | 61 | Left cerebellopontine | Grade3 | 1 | 5 | 219 |
| 5 | female | 82 | Left frontal lobe | Grade4 | 1 | 24 | 190 |
| 6 | female | 56 | Left temporal lobe | Grade4 | 1 | 15 | 186 |
| 7 | female | 40 | Right frontal lobe | Grade2 | 1 | 57 | 166 |
| 8 | male | 63 | Left frontal lobe | Grade4 | 1 | 3 | 244 |
| 9 | female | 41 | Right temporal | Grade4 | 1 | 58 | 258 |
| 10 | female | 50 | Intracranial | Grade4 | 1 | 45 | 267 |
| 11 | male | 63 | Left temporal lobe | Grade4 | 1 | 6 | 205 |
| 12 | male | 48 | Left frontal lobe and left parietal lobe | Grade4 | 1 | 10 | 227 |
| 13 | male | 68 | Right temporal | Grade4 | 1 | 10 | 272 |
| 14 | male | 39 | Right frontal lobe | Grade4 | 1 | 19 | 223 |
| 15 | female | 62 | Left frontal lobe | Grade3 | 1 | 20 | 152 |
| 16 | male | 43 | Left temporal occipital | Grade4 | 1 | 55 | 245 |
| 17 | female | 43 | Right top pillow | Grade4 | 1 | 12 | 244 |
| 18 | male | 48 | Left temporal occipital lobe | Grade3 | 1 | 39 | 236 |
| 19 | male | 69 | Head | Grade4 | 1 | 6 | 168 |
| 20 | female | 72 | Right frontal fundus | Grade4 | 1 | 8 | 216 |
| 21 | female | 62 | Right frontal | Grade2 | 1 | 35 | 171 |
| 22 | male | 70 | Right temporal lobe | Grade3 | 1 | 14 | 187 |
| 23 | male | 73 | Right temporal lobe | Grade4 | 1 | 13 | 138 |
| **Tissue microarray chip5 (19 glioma samples)** | | | | | | | |
| **Patient ID** | **Gender** | **Age (years)** | **Anatomic Site** | **Grade** | **Death** | **Month** | **IHC-score of TP53I13** |
| 1 | male | 74 | Right temporal lobe | Grade3 | 1 | 5 | 117 |
| 2 | female | 59 | Right frontal lobe | Grade3 | 1 | 5 | 167 |
| 3 | male | 50 | Right temporal lobe | Grade4 | 1 | 14 | 185 |
| 4 | male | 54 | Right frontal | Grade3 | 1 | 7 | 198 |
| 5 | female | 58 | Left brain | Grade4 | 1 | 5 | 207 |
| 6 | male | 48 | Right frontal lobe | Grade4 | 1 | 19 | 168 |
| 7 | female | 12 | cerebellum | Grade2 | 1 | 37 | 169 |
| 8 | female | 37 | Left frontal lobe | Grade2 | 1 | 11 | 172 |
| 9 | male | 68 | Left temporal lobe | Grade4 | 1 | 8 | 122 |
| 10 | male | 50 | Right temporal parietal occipital lobe | Grade4 | 1 | 6 | 215 |
| 11 | male | 23 | Left parietal occipital | Grade4 | 1 | 23 | 138 |
| 12 | male | 38 | Right frontotemporal lobe | Grade2 | 1 | 51 | 86 |
| 13 | female | 34 | Right frontal | Grade2 | 1 | 42 | 132 |
| 14 | female | 48 | Left temporal | Grade4 | 1 | 35 | 157 |
| 15 | female | 50 | Right top | Grade4 | 1 | 10 | 159 |
| 16 | male | 48 | Right parietal occipital part | Grade4 | 1 | 10 | 139 |
| 17 | male | 68 | Left temporal | Grade2 | 1 | 10 | 146 |
| 18 | female | 28 | Right frontal lobe | Grade2 | 1 | 8 | 210 |
| 19 | male | 73 | Right temporal | Grade4 | 1 | 10 | 207 |
| **Tissue microarray chip6 (23 glioma samples)** | | | | | | | |
| **Patient ID** | **Gender** | **Age (years)** | **Anatomic Site** | **Grade** | **Death** | **Month** | **IHC-score of TP53I13** |
| 1 | female | 48 | Right frontal part | Grade2 | 0 | 94 | 209 |
| 2 | male | 59 | Left temporal lobe | Grade2 | 0 | 94 | 272 |
| 3 | female | 52 | Right frontal part | Grade2 | 1 | 2 | 213 |
| 4 | female | 71 | Right occipital part | Grade3 | 1 | 4 | 169 |
| 5 | female | 61 | Right frontal parietal | Grade3 | 1 | 2 | 295 |
| 6 | male | 55 | Parietal occipital part | Grade4 | 1 | 15 | 255 |
| 7 | female | 58 | Left temporal | Grade4 | 1 | 12 | 265 |
| 8 | female | 47 | Left temporal | Grade3 | 1 | 10 | 204 |
| 9 | male | 60 | Left frontal part | Grade4 | 1 | 4 | 298 |
| 10 | male | 64 | Right parietal lobe | Grade4 | 1 | 11 | 293 |
| 11 | female | 25 | Right frontal lobe | Grade3 | 1 | 31 | 252 |
| 12 | male | 61 | Head | Grade3 | 1 | 21 | 160 |
| 13 | male | 57 | Right temporal | Grade2 | 1 | 7 | 282 |
| 14 | female | 43 | Right frontal lobe and triangle | Grade2 | 1 | 18 | 204 |
| 15 | male | 50 | Right frontal lobe | Grade4 | 1 | 30 | 256 |
| 16 | female | 54 | Right temporal | Grade3 | 1 | 55 | 232 |
| 17 | male | 27 | Right frontal lobe | Grade3 | 1 | 38 | 284 |
| 18 | male | 50 | Right parietal temporal | Grade3 | 1 | 46 | 206 |
| 19 | female | 34 | Right frontal lobe | Grade4 | 1 | 34 | 277 |
| 20 | male | 66 | Right parietal occipital part | Grade3 | 1 | 30 | 221 |
| 21 | female | 33 | Right frontal lobe | Grade2 | 0 | 95 | 179 |
| 22 | male | 50 | Left temporal | Grade3 | 1 | 39 | 207 |
| 23 | male | 64 | Left parietal lobe | Grade3 | 1 | 20 | 247 |
| **Tissue microarray chip7 (16 glioma samples)** | | | | | | | |
| **Patient ID** | **Gender** | **Age (years)** | **Anatomic Site** | **Grade** | **Death** | **Month** | **IHC-score of TP53I13** |
| 1 | male | 48 | Left frontal lobe | Grade3 | 1 | 9 | 121 |
| 2 | male | 64 | Top right | Grade3 | 1 | 24 | 106 |
| 3 | female | 70 | Left frontal lobe | Grade3 | 1 | 26 | 171 |
| 4 | female | 48 | Head | Grade2 | 1 | 44 | 118 |
| 5 | male | 50 | Superficial left frontal lobe | Grade4 | 1 | 9 | 104 |
| 6 | male | 70 | Left frontal lobe | Grade4 | 1 | 4 | 141 |
| 7 | female | 44 | Head | Grade4 | 1 | 8 | 160 |
| 8 | male | 24 | Left occipital part | Grade3 | 0 | 68 | 101 |
| 9 | male | 71 | Left frontal lobe | Grade3 | 0 | 68 | 174 |
| 10 | female | 68 | Right frontotemporal lobe | Grade4 | 1 | 6 | 128 |
| 11 | male | 53 | Left frontal lobe | Grade4 | 1 | 13 | 156 |
| 12 | male | 52 | Left frontal lobe | Grade3 | 1 | 28 | 178 |
| 13 | male | 18 | Left temporal | Grade3 | 0 | 68 | 116 |
| 14 | male | 28 | Right frontal | Grade4 | 1 | 17 | 119 |
| 15 | male | 50 | Right temporal lobe | Grade3 | 1 | 11 | 178 |
| 16 | female | 58 | Left basal ganglia | Grade3 | 1 | 14 | 173 |
