# Supplementary material for: TP53I13 promotes metastasis in glioma via macrophages, neutrophils, and fibroblasts and is a potential prognostic biomarker
Source: Front Immunol. 2022 Oct 7;13:974346. doi: 10.3389/fimmu.2022.974346 (PMC9585303; doi:10.3389/fimmu.2022.974346)
Supplement: Supplementary file 12 [file Table_2.docx]

Supplementary Table 2

| Characteristics | Univariate analysis | | | | Multivariate analysis | | | |
| --- | --- | --- | --- | --- | --- | --- | --- | --- |
|  | HR | HR.95L | HR.95H | P-value | HR | HR.95L | HR.95H | P-value |
| TP53I13 | 1.509 | 1.380 | 1.650 | < 0.001 | 1.138 | 1.027 | 1.260 | 0.013 |
| PRS_type | 2.256 | 1.923 | 2.647 | < 0.001 | 2.254 | 1.903 | 2.669 | < 0.001 |
| Histology | 4.212 | 3.444 | 5.151 | < 0.001 | 0.834 | 0.531 | 1.308 | 0.429 |
| Grade | 2.777 | 2.416 | 3.192 | < 0.001 | 2.356 | 1.712 | 3.244 | < 0.001 |
| Gender | 1.094 | 0.902 | 1.327 | 0.359 | 1.051 | 0.863 | 1.281 | 0.621 |
| Age | 1.614 | 1.327 | 1.963 | < 0.001 | 1.338 | 1.093 | 1.638 | 0.005 |
| Radiotherapy | 0.919 | 0.727 | 1.160 | 0.476 | 1.009 | 0.789 | 1.291 | 0.941 |
| Chemotharpy | 1.259 | 1.010 | 1.569 | 0.041 | 0.574 | 0.450 | 0.731 | < 0.001 |
| IDH_mutation | 0.328 | 0.269 | 0.399 | < 0.001 | 0.675 | 0.528 | 0.863 | 0.002 |
| 1p/19q codeletion | 0.232 | 0.169 | 0.319 | < 0.001 | 0.375 | 0.267 | 0.528 | < 0.001 |
| MGMTp_methylation | 0.844 | 0.698 | 1.020 | 0.079 | 0.863 | 0.707 | 1.053 | 0.147 |

Univariate and multivariate cox analysis of TP53I13 expression level and different clinical characteristics
