# Supplementary material for: TP53I13 promotes metastasis in glioma via macrophages, neutrophils, and fibroblasts and is a potential prognostic biomarker
Source: Front Immunol. 2022 Oct 7;13:974346. doi: 10.3389/fimmu.2022.974346 (PMC9585303; doi:10.3389/fimmu.2022.974346)
Supplement: Supplementary file 13 [file Table_3.docx]

Supplementary Table 3

| **Characteristics** | **Univariate analysis** | | | | **Multivariate analysis** | | | |
| --- | --- | --- | --- | --- | --- | --- | --- | --- |
|  | **HR** | **HR.95L** | **HR.95H** | **P** | **HR** | **HR.95L** | **HR.95H** | **P** |
| TP53I13 | 1.963 | 1.351 | 2.853 | ＜0.001 | 1.900 | 1.304 | 2.768 | 0.001 |
| Age(≤60） | 1.923 | 1.345 | 2.747 | ＜0.001 | 1.569 | 1.092 | 2.255 | 0.015 |
| Gender | 1.524 | 1.069 | 2.171 | 0.020 | 1.419 | 0.992 | 2.030 | 0.055 |
| IDH(m/w) | 0.856 | 0.544 | 1.348 | 0.503 |  |  |  |  |
| Type | 0.974 | 0.861 | 1.103 | 0.682 |  |  |  |  |
| Grade | 0.986 | 0.980 | 0.991 | ＜0.001 | 0.987 | 0.982 | 0.993 | ＜0.001 |

Univariate and multivariate cox analysis of TP53I13 and different clinical characteristics based on samples from Nantong University Affiliated Hospital
